# Inhibition of lobuloalveolar development by FOXC1 overexpression in the mouse mammary gland

**DOI:** 10.1038/s41598-017-14342-8

**Published:** 2017-10-25

**Authors:** Bowen Gao, Ying Qu, Bingchen Han, Yoshiko Nagaoka, Makoto Katsumata, Nan Deng, Shikha Bose, Liting Jin, Armando E. Giuliano, Xiaojiang Cui

**Affiliations:** 1 Department of Surgery, Samuel Oschin Comprehensive Cancer Institute, Cedars-Sinai Medical Center, Los Angeles, CA, 90048, USA; 2 Department of Biomedical Sciences, Samuel Oschin Comprehensive Cancer Institute, Cedars-Sinai Medical Center, Los Angeles, CA, 90048, USA; 3 Biostatistics and Bioinformatics Research Center, Samuel Oschin Comprehensive Cancer Institute, Cedars-Sinai Medical Center, Los Angeles, CA, 90048, USA; 4 Department of Pathology, Samuel Oschin Comprehensive Cancer Institute, Cedars-Sinai Medical Center, Los Angeles, CA, 90048, USA; 5 Department of Breast Surgery, Hubei Cancer Hospital, Wuhan, Hubei, 430079, China

## Abstract

The forkhead box transcription factor FOXC1 plays a critical role in embryogenesis and the development of many organs. Its mutations and high expression are associated with many human diseases including breast cancer. Although FOXC1 knockout mouse studies showed that it is not required for mammary gland development during puberty, it is not clear whether its overexpression alters normal mammary development *in vivo*. To address this question, we generated transgenic mice with mammary-specific FOXC1 overexpression. We report that transgenic FOXC1 overexpression suppresses lobuloalveologenesis and lactation in mice. This phenotype is associated with higher percentages of estrogen receptor-, progesterone receptor-, or ki67-positive mammary epithelial cells in the transgenic mice at the lactation stage. We also show that expression of the Elf5 transcription factor, a master regulator of mammary alveologenesis and luminal cell differentiation, is markedly reduced in mammary epithelial cells of transgenic mice. Likewise, levels of activated Stat5, another inducer of alveolar expansion and a known mediator of the Elf5 effect, are also lowered in those cells. In contrast, the cytokeratin 8-positive mammary cell population with progenitor properties is elevated in the transgenic mice at the lactation stage, suggesting inhibition of mammary cell differentiation. These results may implicate FOXC1 as a new important regulator of mammary gland development.

## Introduction

Mammary gland development is a highly dynamic and complex physiological process in mammals that involves periodic cycles of mammary epithelium proliferation, differentiation, apoptosis, and morphogenesis throughout a female’s life^1^. Generally, postnatal development of the mammary gland comprises several distinct stages. The mammary ducts elongate through the entire mammary fat pad at puberty and branches out in the mature virgin. During pregnancy, alveologenesis is stimulated by prolactin and progesterone. Alveolar structures further differentiate at the lactation stage to produce milk. After weaning, the gland involutes and undergoes cell death and remodeling of the epithelial compartment. An assortment of microenvironmental cues and intracellular signaling networks that include critical transcription factors act in concert to govern mammary tissue remodeling during the developmental stages and recurring physiologic changes induced by reproductive hormones^2,3^. In addition, the mammary gland epithelium is constantly maintained and replenished by a small subset of epithelial cells called mammary stem cells and progenitor cells. Although our knowledge of mammary gland biology has taken a quantum leap in the last two decades and has advanced our understanding and treatment of breast diseases, the in-depth molecular mechanisms underlying mammary gland development awaits to be elucidated.

The transcription factor forkhead box C1 (FOXC1), a member of the forkhead box protein family, has been shown to play a key role in mouse embryonic development^4,5^. FOXC1 knockout mice die at birth with extensive abnormalities including hemorrhagic hydrocephalus, open eyelids, and striking skeletal defects^6^. Human brain imaging studies coupled with genetic analysis show that deletions or duplications of the *FOXC1* gene are associated with cerebellar and posterior fossa malformations^7^, and these abnormalities can be caused by FOXC1 loss-induced cerebellar growth inhibition^8^. FOXC1 is also required for cardiovascular development. It acts in cooperation with FOXC2 to promote the formation of blood vessels in the embryonic heart^9^. Both genes are required for arterial specification during vascular development and the morphogenesis of the cardiac outflow tract^10,11^. Moreover, FOXC1 is essential for somitogenesis in both mouse and zebrafish models^9,12^. Besides its role in embryonic development, emerging findings indicate that FOXC1 is also involved in cancer development and progression. Of note, FOXC1 is specifically expressed in basal-like breast cancer (BLBC)^13,14^, but not in other breast cancer subtypes, and its overexpression predicts a poor prognosis^14^. Thus, FOXC1 has been proposed to be a diagnostic and prognostic biomarker for BLBC^15^. In addition to its known effects in cell proliferation and migration found in both normal and cancer cells, FOXC1 also regulates many other breast cancer cell functions, including epithelial-mesenchymal transition, invasion^14,16^, and breast cancer stem cell properties^17^. FOXC1 overexpression has been recently found to be associated with poor prognosis in other cancers, such as acute myeloid leukemia (AML)^18^ and hepatocellular carcinoma^19^.

In light of its emerging role in normal development and breast cancer tumorigenesis, the present study was designed to explore whether FOXC1 transgene expression affects mammary gland development *in vivo*. For this purpose, we generated a transgenic mouse model that expresses the human *FOXC1* cDNA under the transcriptional control of the mouse mammary tumor virus (MMTV) long terminal repeat and examined the effects of ectopic FOXC1 transgene expression on mammary gland development. Compared with the wildtype controls, the FOXC1 transgenic mice were characterized by defective alveologenesis and milk production during the lactation stage. This phenotype is accompanied with drastically reduced levels of the Elf5 transcription factor and activated STAT5, which are well-established alveologenesis inducers with high expression in the mammary gland during lactation. Furthermore, mammary progenitor cell populations were increased by FOXC1 overexpression. These results may provide insight into how transgenic FOXC1 expression disrupts mammary gland development *in vivo*.

## Results

### Generation of MMTV-FOXC1 transgenic mice

**Figure 1:**
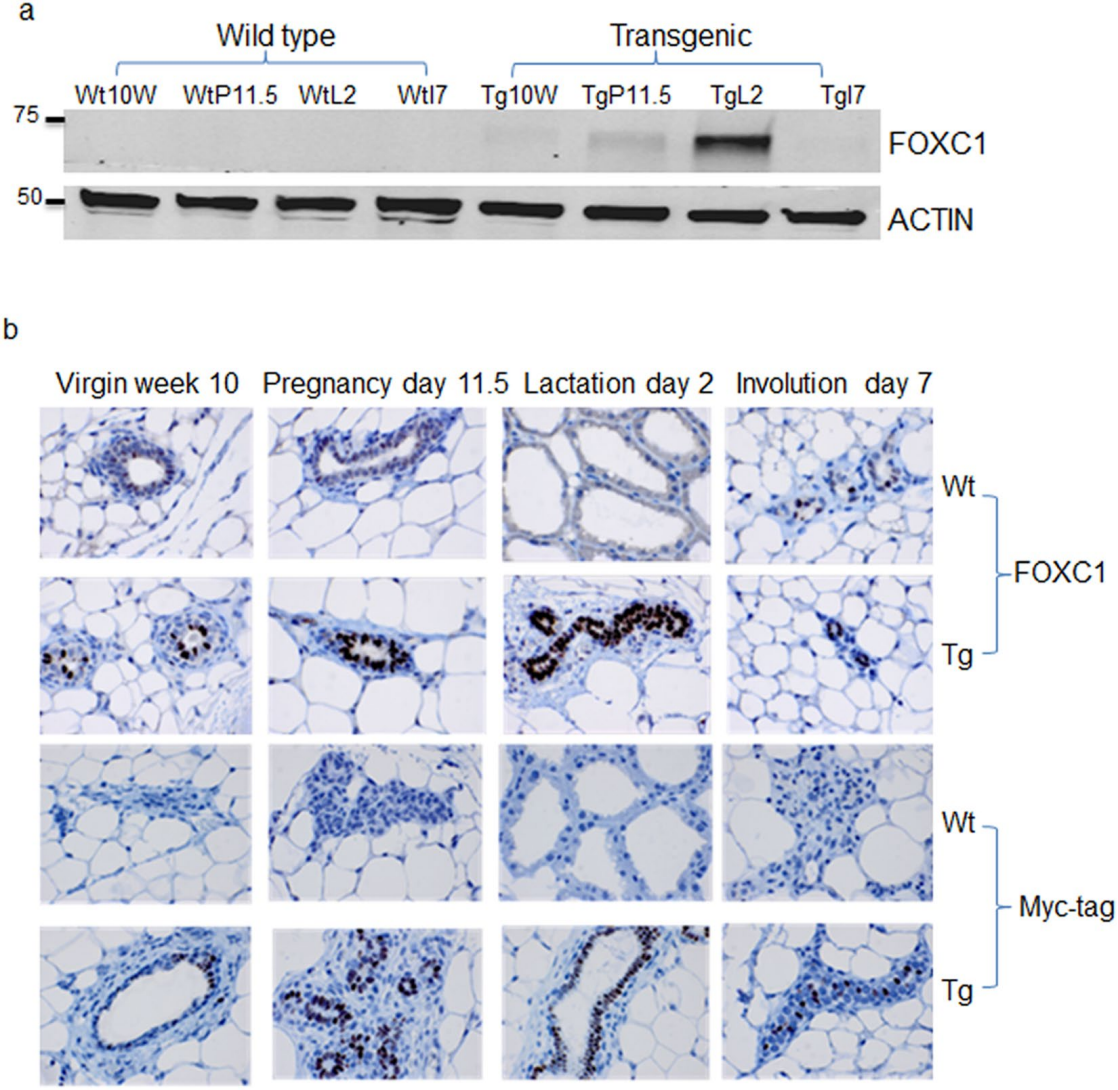
Generation and characterization of MMTV-FOXC1 transgenic mice. (**a**) FOXC1 expression by western blotting in mammary gland tissue collected at different time points: virgin week 10, pregnancy day 11.5, lactation day 2, and involution day 7 from the wildtype and transgenic mice. For the full-length image, see Figure S5 in the Supplementary Information. (**b**) Analysis of FOXC1 and Myc-tag by immunohistochemistry in mammary gland tissue collected at the above different time points from the wildtype and transgenic mice. Magnification, ×60. See also Figure S1.

To examine the effect of FOXC1 on normal mammary cells *in vivo*, we used the mouse mammary tumor virus (MMTV) promoter/enhancer to drive the ectopic expression of a Myc-tagged FOXC1 transgene in the mouse mammary gland. The MMTV-FOXC1 construct (Figure S1a) was micro-injected into the fertilized eggs of FVB/NJ mice. The transgenic founders were identified by PCR analysis using genomic DNA extracted from the tail (Figure S1b). They were then bred with FVB/NJ wildtype mice to ensure that the transgene was passed on to their progeny. The transgene expression was assessed by western blot analysis of mammary gland tissues, which was collected at different time points: virgin week 10, pregnancy day 11.5, lactation day 2, and involution day 7 from wildtype and transgenic mice. As presented in Fig. 1a, consistent with the known hormonal responsiveness of the MMTV-promoter, FOXC1 protein levels were barely detectable in the mammary tissue at the virgin stage and moderately induced during pregnancy. However, it was highly expressed during the lactation stage in the transgenic mice compared to the wildtype mice. FOXC1 transgene expression in luminal epithelial cells, not myoepithelial cells, was detected by immunohistochemistry with FOXC1 and Myc-tag antibodies (Fig. 1b). Furthermore, the tissue specificity of Myc-tagged FOXC1 expression was analyzed by immunohistochemistry in heart, liver, spleen, lung, kidney, intestine, pancreas, ovary, brain, and stomach tissues from the wildtype and transgenic mice. As shown in Figure S1c, the FOXC1 transgene was not detected in these tissues as opposed to mammary tissue. Taken together, these results indicate that the MMTV-FOXC1 transgenic mice express the transgene specifically in the luminal epithelium of the mammary gland.

### FOXC1 overexpression inhibits lobuloalveologenesis

**Figure 2:**
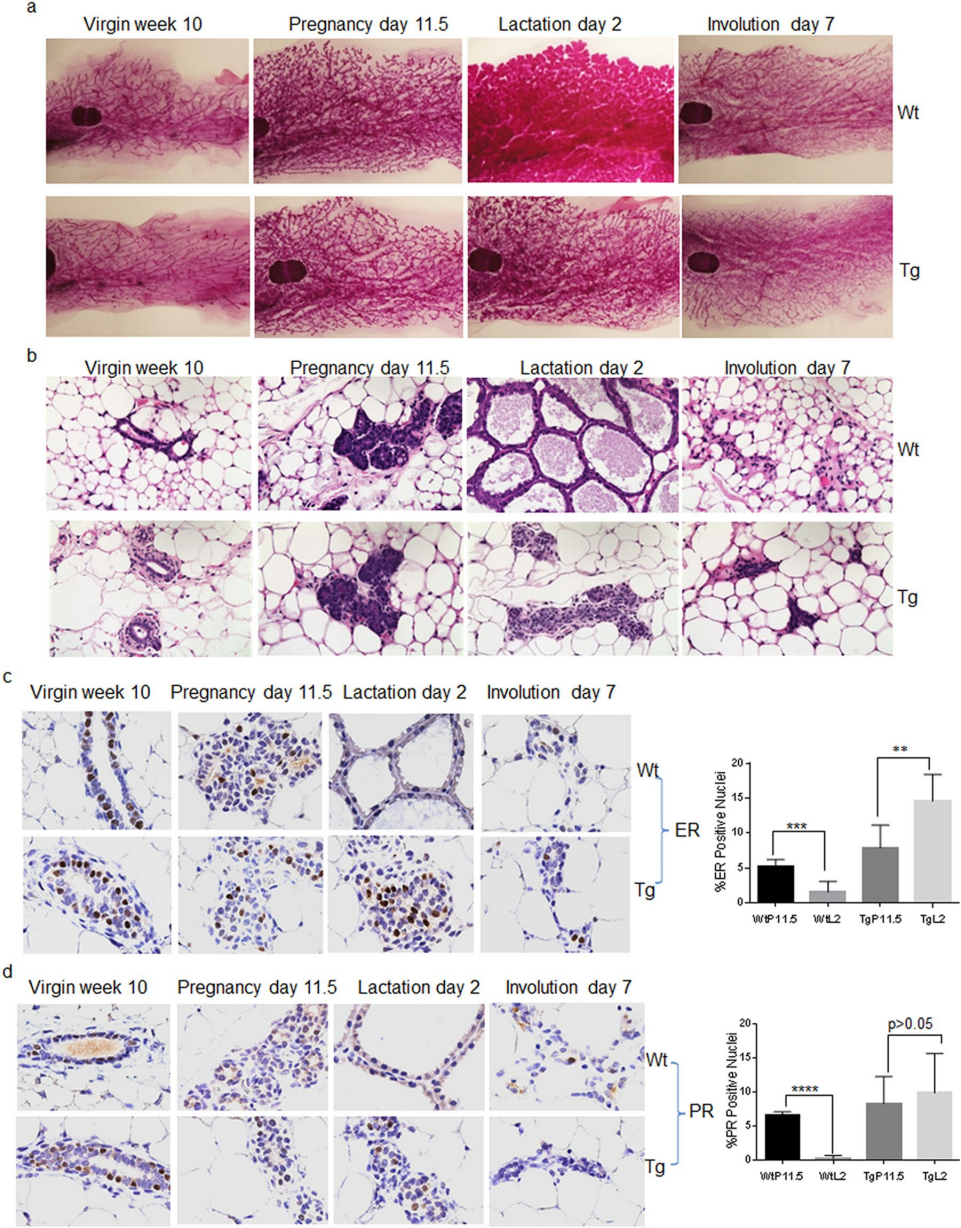
FOXC1 overexpression inhibits lobuloalveologenesis. (**a**) Whole mount staining analysis of mammary gland tissue collected at different time points: virgin week 10, pregnancy day 11.5, lactation day 2, and involution day 7 from the wildtype and transgenic mice. Magnification, ×1. (**b**) Hematoxylin eosin staining of mammary gland tissue collected at the same time points. Magnification, ×40. (**c**) and (**d**) Immunohistochemistry of ER and PR in mammary gland tissue collected at the same time points. Magnification, ×60. Comparison of the percentages of ER+ and PR+ nuclei at pregnancy day 11.5 and lactation day 2 from the wildtype and transgenic mice is graphed. The percentages were calculated by comparing stained nuclei to total nuclei in epithelial cells. The bar graph indicates mean ± SD (n = 5). **p < 0.01. ***p < 0.001. ****p < 0.0001. See also Figure S2.

We next characterized the phenotypes of six transgenic lines with readily detectable Myc-tagged FOXC1 protein expression. Surprisingly, during the breeding of transgenic female mice, it was noted that their pups always died on postnatal day 1 or 2. Examination of the dead pups indicated that there was no milk in their stomachs. When the newly born pups were nursed by wildtype foster mothers, they exhibited a normal appearance, growth rate, and physiological function (data not shown). This phenomenon was observed consistently in a total of 41 MMTV-FOXC1 transgenic female mice, none of which could support their offspring. Considering this observation, we set out to determine whether mammary morphogenesis was affected by FOXC1 transgene expression using whole mount carmine staining of isolated mammary glands. As shown in Fig. 2a, lobuloalveolar morphogenesis at the lactation stage was severely impaired in the transgenic mice compared to the wildtype mice, while no obvious differences were found in the other mammary development stages. In agreement, haematoxylin eosin staining also demonstrated the absence of well-defined alveolar structures at the lactation stage in the transgenic mice when compared to the wildtype controls (Fig. 2b). These findings suggest that lobuloalveolar formation of the mouse mammary gland is repressed by FOXC1 overexpression.

We reasoned that expression of established critical regulators of lobuloalveologenesis might be changed in FOXC1 transgenic mice. It is well-known that estrogen and progesterone play fundamental roles in mammary gland differentiation^1,20^. Their receptors, estrogen receptor (ER) and progesterone receptor (PR), are required for ductal and alveolar morphogenesis^21,22^. Thus, we investigated whether the expression of ER and PR is affected by FOXC1 transgene expression. Immunohistochemical analysis showed that the percentages of ER+ and PR+ mammary epithelial cells in the total mammary epithelial cell population dropped substantially from the pregnancy stage to the lactation stage in wildtype mice as reported previously^20^, whereas this expression pattern was reversed in transgenic mice (Fig. 2c and d). A 3.3-fold decrease of ER+ cells in wildtype mice versus a 1.85-fold increase in transgenic mice were detected. Similarly, a 6.1-fold decrease of PR+ cells in wildtype mice versus a statistically non-significant increase in transgenic mice were observed. There were no detectable differences in ER and PR staining between wildtype and transgenic mice at the other three developmental stages. We also performed immunohistochemistry of Ki67, a cellular marker for proliferation. As expected, wildtype controls displayed reduced Ki67+ staining at the lactation stage when mammary cells undergo lactogenesis, whereas the percentages of Ki67+ mammary cells were similar between pregnancy day 11.5 and lactation day 2 in transgenic mice (Figure S2). Taken together, these results suggest that FOXC1 overexpression may alter the proportions of ER-, PR-, and Ki67-expressing mammary epithelial cells during the lactation stage, consistent with the whole mount staining showing that the FOXC1 transgene disrupts lobuloalveolar development.

### Elf5 expression is suppressed in mammary epithelial cells of transgenic mice at lactation

**Figure 3:**
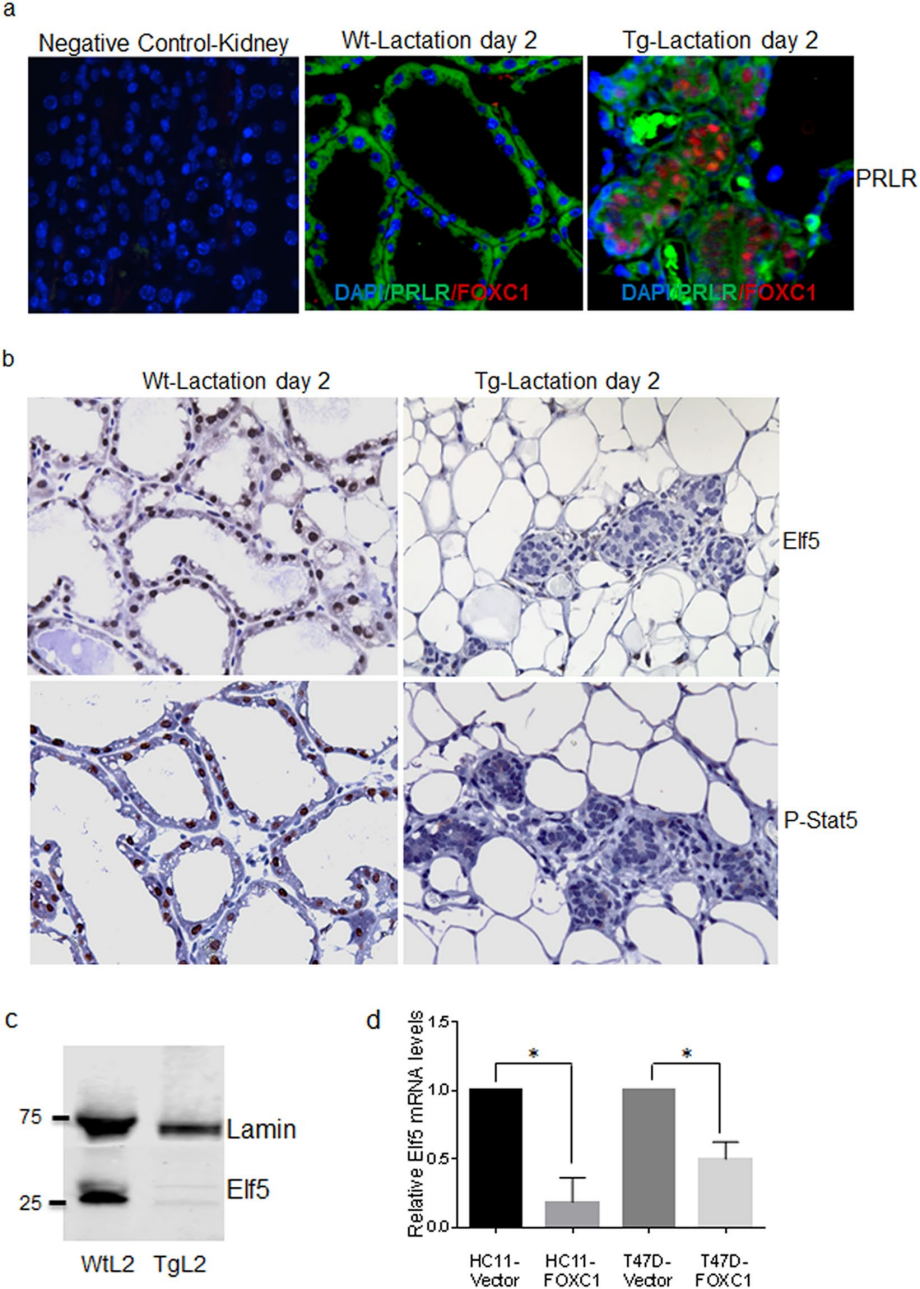
Elf5 expression is suppressed in mammary epithelial cells overexpressing FOXC1. (**a**) Immunofluorescence staining of FOXC1 and PRLR in the mammary epithelium from the wildtype and transgenic mice at lactation day 2. Magnification, ×40. (**b**) Immunohistochemistry of Elf5 and p-Stat5 in the nuclei of the mammary epithelium from the wildtype and transgenic mice at lactation day 2. Magnification, ×40. (**c**) Elf5 expression by western blotting in mammary gland tissue, collected at lactation day 2 from the wildtype and transgenic mice. For the full-length image, see Figure S5 in the Supplementary Information. (**d**) qRT-PCR analysis of Elf5 mRNA expression in vector- and FOXC1-transfected HC11 mouse epithelial cells and T47D human breast cancer cells. The bar graph indicates mean ± SD (n = 3). *p < 0.05. See also Figure S3.

Prolactin and its receptor PRLR are key regulators of lactogenesis. They crosstalk with estrogen and progesterone signaling to exert their effects on the mammary gland^23^, particularly in the alveolar epithelium^22^. We next asked whether PRLR expression was affected because this receptor is a rate-limiting factor for the response of the mammary gland to prolactin and its expression is dynamically regulated by hormones like estrogen during mammary gland development^24^. Immunofluorescence staining showed that PRLR signals were comparable in the mammary glands of wildtype and transgenic mice at lactation day 2 (Fig. 3a). Real-time RT-PCR analysis also demonstrated that FOXC1 overexpression did not induce significant changes of PRLR expression (Figure S3a). However, the expression of the Elf5 transcription factor, which is a key target and mediator of alveolar switch hormones, such as progesterone and prolactin, and regarded as a master regulator of the lobuloalveolar process^25–28^, was found to be markedly suppressed in transgenic mice compared to wildtype controls at lactation day 2 as shown by immunohistochemistry and western blotting (Fig. 3b and c). This result was confirmed by real-time RT-PCR analysis of Elf5 mRNA levels (Figure S3b). RNA-Seq analysis of mammary cells from lactation day 2 also showed a dramatic decrease of Elf5 in transgenic mice (Table S1). In contrast, no discernable changes of Elf5 expression were observed between wildtype and transgenic virgin and pregnant mammary glands as shown by immunohistochemistry (Figure S3c). To further determine the effect of FOXC1 on Elf5 levels, we overexpressed FOXC1 in HC11 mouse mammary epithelial cells and T47D human breast cancer cells. As illustrated in Fig. 3d, FOXC1 overexpression significantly downregulated Elf5 mRNA levels. Because there is one potential FOXC1-binding site (GTAAATCAA, at −1360 bp) with the core consensus sequence GTAAA based on transcription factor site analysis of the Elf5 promoter region, we tested whether FOXC1 directly binds to the promoter region containing the site using chromatin immunoprecipitation (ChIP) assays. As presented in Figure S3d, FOXC1 antibody did not pull down the Elf5 promoter fragment containing the predicted binding site, suggesting that FOXC1 may reduce Elf5 in mammary epithelial cells either by other upstream binding sites or by an indirect mechanism.

Given that Stat5 is a direct transcriptional target and effector of Elf5 and is required for alveolar fate commitment and lactogenesis^25,29^, we examined Stat5 activation in the mouse mammary tissue by immunohistochemistry. As illustrated in Fig. 3b (lower panel), levels of activated Stat5 indicated by p-Stat5 (Tyr 694) were diminished in mammary cells from transgenic mice at lactation day 2 compared to wildtype mice. Of note, pathway analysis of the differentially expressed genes identified by RNA-Seq in these cells suggest that fatty acid metabolism, PPAR, cytokine, chemokine, inflammation, and NF-κB pathways are significantly altered in transgenic mice based on analysis of differential gene expression (Table S2), which is consistent with lactogenesis blockage. Given that Stat5 has been reported to be involved in some of these differentially regulated pathways^30–32^, reduced p-Stat5 levels could be a cause or effect of these changes. We further examined p-Stat5 in HC11 mouse mammary cells overexpressing FOXC1. Immunoblotting showed that p-Stat5 levels were decreased in these cells compared with vector controls (Figure S3e). Taken together, these results suggest that FOXC1 overexpression impedes mammary lobuloalveolar differentiation and correlates with lower Elf5 and p-Stat5 levels in transgenic mammary glands at the lactation stage.

### The luminal progenitor population is increased in transgenic mice at lactation

**Figure 4:**
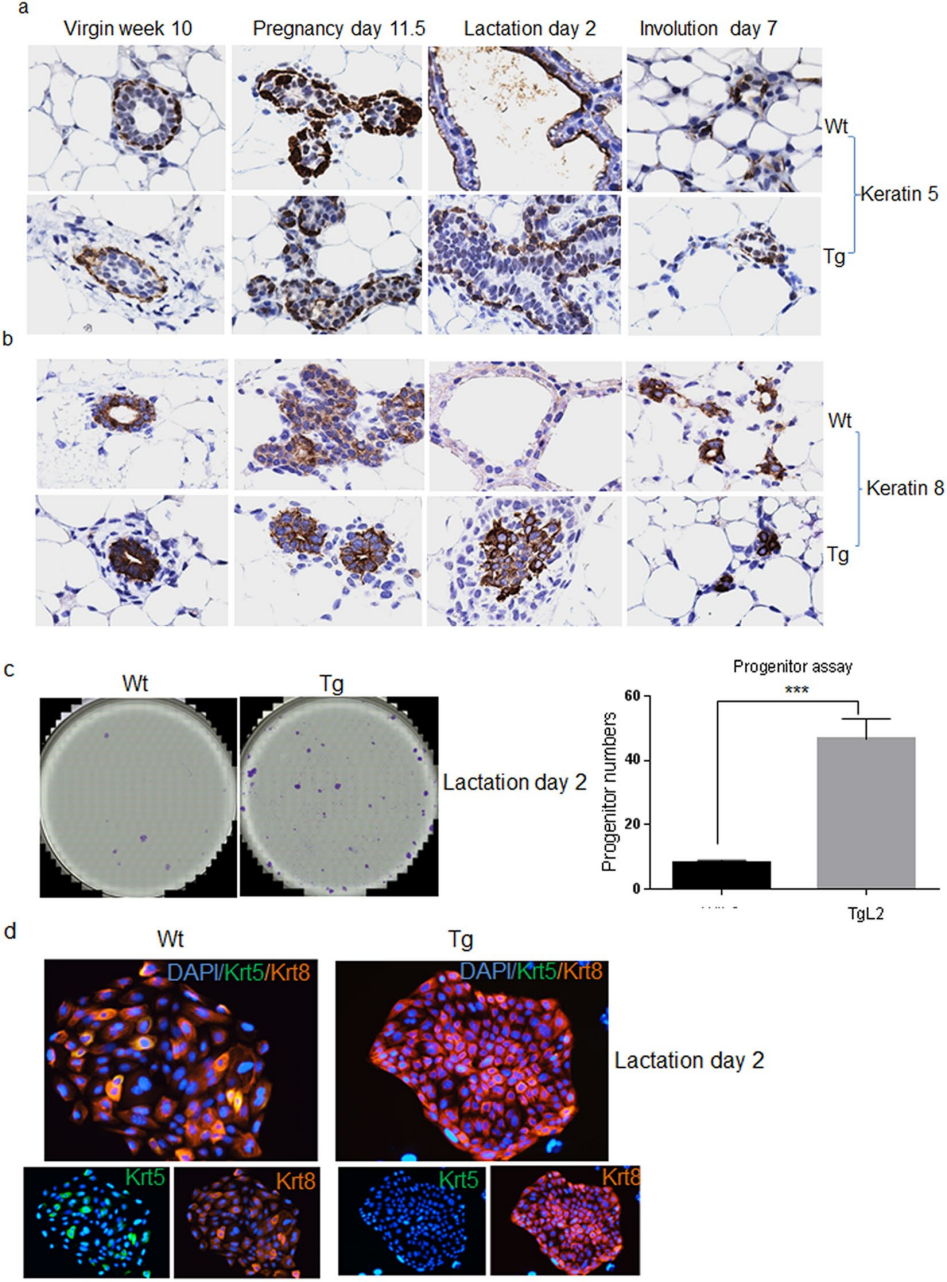
The luminal progenitor population is increased in transgenic mice at lactation. Immunohistochemistry of CK5 (**a**) and CK8 (**b**) in mammary epithelial cells collected at different time points: virgin week 10, pregnancy day 11.5, lactation day 2, and involution day 7 from the wildtype and transgenic mice. Magnification, ×40. (**c**) Colony-forming assays of epithelial cells from the wildtype and transgenic mice at lactation day 2. Images were obtained with magnification ×10. The bar graph indicates mean ± SD (n = 3). ***p < 0.001. (**d**) Immunofluorescence staining of CK5 and CK8 in the progenitor colonies of mammary epithelial cells from the wildtype and transgenic mice at lactation day 2. Magnification, ×40. See also Figure S4.

It is known that a normal intact luminal epithelium is required for complete lobuloalveolar development^28^. To assess whether FOXC1 affects the differentiation of luminal epithelium to alveolar epithelium, we performed immunohistochemical staining of cytokeratin 8 (CK8) and CK5, established mammary luminal and basal epithelial cell markers, respectively. As shown in Fig. 4a, CK5 presented a similar staining pattern in the mammary glands at different stages of wildtype and transgenic mice, which is probably in part due to that the MMTV promoter is not active in the basal compartment. As expected, CK8 staining in wildtype mice was prominent in mammary tissues from the virgin, pregnancy, and involution stages, but was markedly reduced at the lactation stage^33^. In stark contrast, strong CK8 staining was observed in transgenic mice at the lactation stage (Fig. 4b), whereas its staining at the other stages was similar to wildtype mice. These data suggest that the alveolar differentiation of luminal epithelial cell is impaired by FOXC1 overexpression. Given that Elf5 deletion in the mammary gland leads to accumulation of the luminal progenitor population^27,34^, we proceeded to test whether the mammary progenitor pool could be augmented by the FOXC1 transgene expression. As shown in Fig. 4c, colony-forming assays in low-cell density adherent culture using isolated mammary epithelial cells from the lactation stage showed that the number of mammary progenitors, which produced discrete colonies, was markedly increased in transgenic mice compared with wildtype mice at the lactation stage. In contrast, no differences in progenitor numbers at the virgin and pregnancy stages were observed (Figure S4a), consistent with concomitant low FOXC1 transgene expression. Of note, when comparing the pregnancy and lactation stages, the progenitor number decreased in wildtype mice as reported, but increased 2 fold in transgenic mice. Furthermore, immunofluorescence staining revealed that the progenitor-derived colonies from wildtype mice contained CK8+ and CK5+ CK8+ cells, whereas the colonies from transgenic mice were largely comprised of CK8+ cells (Fig. 4d). To consolidate our studies, we performed colony formation assays in HC11 cells. As shown in Figure S4b, FOXC1 overexpression increased the colony-forming ability of HC11 cells. Taken together, these results indicate that the lactation failure in transgenic mice is associated with an increased mammary luminal cell population with progenitor properties.

## Discussion

In the present study, we show that the mammary gland-specific FOXC1 overexpression mouse model displays a distinct phenotype of defective lobuloalveologenesis and lactogenesis, accompanied with a reduction in the levels of the two master regulators of lobuloalveolar development, Elf5 and activated Stat5, and an increase in mammary cells with luminal progenitor properties. It is possible that these protein and progenitor cell population changes may be a consequence of the lactation failure in transgenic mice. Our results are consistent with a previous report showing that FOXC1 expression is elevated in the mouse mammary luminal progenitor population^35^. In that study, FOXC1 was found to be unnecessary for mammary gland development at puberty when using orthotopic transplantation of FOXC1 knockout mammary anlagen. One explanation is the potential compensatory effect of FOXC2 and other FOX family members. Our result suggests that transgenic FOXC1 expression can disrupt mammary gland development.

One interesting observation of the MMTV-FOXC1 transgenic mice is a higher percentage of ER+ and PR+ mammary cells in the lactation stage compared with the pregnancy stage versus the diminished ER+ and PR+ cell numbers during lactation in wildtype mice. Generally, proliferation is incompatible with terminal differentiation, and down-regulation of ER and PR expression during lactation may be necessary to achieve lactogenic differentiation^36^. Thus, increase and maintenance of ER+ and PR+ cells during lactation may reflect impaired mammary gland differentiation due to FOXC1 overexpression. Of note, ER expression and activity can be suppressed by Elf5 in mammary cells^37^. It is thus possible that the drastic decrease of Elf5 expression in MMTV-FOXC1 mice may contribute to the increase of ER+ cells in the mammary gland at the lactation stage.

Another notable finding from our study is the reduction of Elf5 expression in transgenic mammary glands with FOXC1 overexpression. Elf5 dictates lobuloalveolar morphogenesis and mammary progenitor cell differentiation toward the secretory phenotype^34^. In light of our cell line data suggesting that Elf5 might act downstream of FOXC1, it is speculative that Elf5 may play a role in the effect of FOXC1 on lactogenesis. Stat5 is a direct downstream target of Elf5 and is a key mediator of other pathways involved in mammary development^38^. Because PR and PRLR expression was not reduced in transgenic mice, the decrease of p-Stat5 levels may be attributed, at least in part, to suppressed Elf5 expression and/or an altered cellular signing network comprising fatty acid metabolism and other pathways. Given that Elf5 deficiency in mammary cells causes expansion of luminal progenitors whereas its overexpression abolishes the progenitor pool and induces the differentiation of progenitors toward milk secretion, the marked reduction of Elf5 levels may implicate its role in the FOXC1-induced effect on mammary gland development and mammary progenitor cells. Moreover, Elf5 inhibits stem-like traits in breast cancer cells^39^. In contrast, FOXC1 has been reported to be essential for maintaining stem cell properties in normal and cancer cells as well as the microenvironment^17,40,41^. Further investigation of the underlying regulatory mechanism between the two genes may implicate the downregulation of Elf5 as a potential pivotal effector of FOXC1 function in mammary cells.

One clinical implication of our study lies in the mechanism of BLBC development. It is now widely accepted that BLBC originates from luminal epithelial progenitors and not from basal stem cells^42^. Given that FOXC1 has emerged as a specific diagnostic and prognostic biomarker for BLBC^14,15^ and that it induces luminal progenitor populations, overexpression of FOXC1 may cooperate with other breast cancer-associated genes or pathways to govern BLBC development. Moreover, FOXC1 transgenic mice may serve as a clinically relevant model, together with other breast cancer mouse models, to address the poorly understood mechanism of BLBC tumorigenesis and progression.

Although the MMTV promoter has been widely used to generate transgenic mouse models, which are instrumental in revealing the genetics and biology of mammary gland and breast cancer development, it has limitations. Its activity depends on hormones and genomic integration sites and has leaky expression in non-mammary tissues. One shortcoming of our current model is that we could not determine what effects FOXC1 overexpression would impose at earlier stages of mammary gland development when the promoter is not active. It is also desirable to explore whether FOXC1 overexpression in different mammary cell populations will give rise to similar phenotypes exhibited in MMTV-FOXC1 mice. Another unresolved question is whether the transgenic mammary gland with strong FOXC1 transgene induction at early lactation resembles a wildtype gland at later pregnancy stages. We hope to address these issues using other FOXC1 transgenic mouse models.

## Methods

### Reagents

All chemicals were purchased from Sigma-Aldrich (St. Louis, MO, USA) unless otherwise stated. Dulbecco’s Modified Eagle Medium (DMEM) and DMEM/F12 were purchased from Thermo Scientific (Waltham, MA, USA). 0.25% Trypsin EDTA was from Corning (Houston, TX, USA). Penicillin-Streptomycin Solution was from Fisher (Houston, TX, USA). Gentle collagenase was purchased from Stem Cell Inc. (Vancouver, BC, Canada).

### Generation of MMTV- FOXC1 transgenic mice

All animal procedures were conducted in accordance with the NIH Guide for the Care and Use of Laboratory Animals and were approved by the Institutional Animal Care and Use Committee of Cedars-Sinai Medical Center. FVB/NJ mice purchased from The Jackson Laboratory (Bar Harbor, ME, USA) were housed in a temperature- and light-controlled environment and fed with standard chow diet *ad libitum*. To generate transgenic mice, a linearized XbaI-SalI fragment of the MMTV-FOXC1 transgene was microinjected into the pronucleus of one-cell embryos collected from FVB/NJ mice. Transgene-positive pups (founder mice (F0)) were identified by PCR of genomic DNA extracted from the toe or tail biopsies. PCR was performed using a set of primers (Forward 5′-AGCAGCAGAACTTCCACTC-3′ and Reverse 3′-GATAGAATTAAACCTTATC-5′), KAPA Mouse DNA extraction Kit (75 °C for 10 min, 95 °C for 5 min, and 45 °C for 1 min) and KAPA 2 G Fast HotStart Genotyping PCR Kit (KAPA Biosystems, Wilmington, MA, USA) according to the following PCR program: 95 °C for 3 min followed by 35 cycles of temperature steps (95 °C for 30 s, 60 °C for 30 s, 72 °C for 45 s), 72 °C for 2 min. Western blot analysis was performed to test the expression level of MMTV-FOXC1 in mammary tissue lysates from different developing stages of F1 transgenic mice which were generated by breeding F0 and FVB/NJ mice. Two transgenic lines containing the highest expression of MMTV-FOXC1 were chosen for further study.

### Tissue collection

The day of the vaginal plug appearance was counted as pregnancy day 0.5. For mammary gland lactation, the day of delivery was considered as day 1. For involution, pups were removed (day 1) to induce the involution. When the number 4 mammary glands were collected for RNA analysis and progenitor assays, the lymph nodes were removed before digestion.

### Quantitative RT-PCR

RNA was extracted from mammary gland tissues using RNeasy Mini kit then was reverse-transcribed using QuantTect Reverse Transcription Kit (Qiagen, Valencia, CA). Transcript levels for targets related to Elf5 and PRLR were measured using the quantitative real-time iQ SYBR Green Supermix kit (Bio-Rad, Hercules, CA), and samples were normalized to endogenous GAPDH. Primer sequences are: Mouse long-form PRLR (5′-TGAGGACGAGCCGCTAATG-3′, 5′-GGTGTGTGGGTTTAACACCTTGA-3′), Mouse Elf5 (5′-TGGACTCCGTAACCCATAGC ACCT-3′, 5′-ATTGCTTAAGGGCTGATGGCATCG), Human Elf5 (5′-CAAGCATGTCACA GTCATAG, 5′-GTCAA CCCGCTCACCAATTC-3′).

### Western blot analysis

Proteins were extracted from mouse mammary tissue which was homogenized with RIPA lysis buffer (Sigma-Aldrich) and then centrifuged 20 min twice at 13,000 rpm. Protein concentration was determined by the BCA Protein Assay Kit (ThermoFisher, Waltham, MA). Proteins (50 ug) were run on 4–20% gradient gels and transferred onto PVDF membranes using a Trans-Blot Turbo transfer pack and Trans-Blot Turbo transfer system (Bio-Rad). Membranes were blocked with Odyssey blocking buffer (LI-COR) and incubated with primary antibodies overnight at 4 °C. The primary antibodies were FOXC1 (1:500, sc-21394, Santa Cruz), Actin (1:500, sc-1616, Santa Cruz), Elf5 (1:200, sc-9645, Santa Cruz), and Lamin A/C (1:500, sc-7292, Santa Cruz), p-Stat5 (1:200, 4322 s, Cell signaling), Stat5(A-9) (1:200, sc-74442, Santa Cruz).

### Mammary gland whole mount preparation

Mammary gland whole mount staining was performed following the standard procedure. In brief, both #4 inguinal mammary gland fat pads of FVB mice and MMTV-FOXC1 transgenic mice were excised at virgin week 10, pregnancy day 11.5, lactation day 2, and involution day 7, then were mounted on Superfrost slides (ThermoFisher), and fixed in ethanol/acetic acid solution (75% ethanol, 25% glacial acetic acid) overnight at room temperature. The fixed glands were washed in 70% ethanol for 15 min and rinsed in distilled water for 10 min (twice). The mammary glands were stained overnight at 4 °C in Carmine Alum (1 g carmine and 2.5 g aluminum potassium sulfate in 500 ml water) and were then dehydrated progressively in 70–95–100% ethanol, cleared in Toluene followed by methyl salicylate. Mammary gland whole mounts were photographed using a dissecting microscope from Carl Zeiss (Objective Plan S 1.0x FWD 81 mm).

### Immunohistochemistry

The #4 mammary glands from wildtype control mice and MMTV-FOXC1 transgenic mice were fixed in formalin (10%) and paraffin-embedded by the Cedars-Sinai Medical Center pathology lab. Sectioning and H&E staining were also performed. Immunohistochemistry was conducted using Vectastain ABC Kit (Vector laboratories) and ImmPACT DAB Kit (Vector). Sections were deparaffinized and rehydrated by xylene and gradient ethanol, respectively. An antigen retrieval method using microwave pretreatment and 0.01 M sodium citrate buffer (pH6) was used for all antibodies (Vector laboratories, Burlingame, CA). Primary antibodies were FOXC1 (1:100, sc-21394, Santa Cruz), Myc-Tag (1:100, #2272, Cell Signaling), ER (1:100, sc-542 Santa Cruz), PR (1:100, sc-538 Santa Cruz), Ki67 (1:100, 790–4286, Roche Diagnostics), Elf5 (1:100, sc-9645, Santa Cruz), Keratin 5 (1:100, 905501, Biolegend), Keratin 8 (1;100, DSHB), p-Stat5(1:100,9359 s, Cell Signaling). The signal was visualized using the VECTASTAIN ABC Systems (Vector laboratories). Counterstaining was performed with Mayer’s hematoxylin (Sigma). The percentages were calculated by comparing stained nuclei to total nuclei in epithelial cells.

### Immunofluorescence staining

Slides underwent de-paraffinization and were rehydrated, then blocked with 5% BSA, incubated with primary antibodies (FOXC1, 1:100, Onconostic Technologies; PRL-R, 1:100, sc-30225, Santa Cruz; Keratin 5, 1:100, 905501, Biolegend; Keratin 8, 1:100, DSHB) overnight at 4 °C and secondary antibodies (Alexa Fluor 488 and 594; 488 and 546, Life Technologies) for 1 hour at room temperature. The nuclei were stained with DAPI (Life Technologies). Images were acquired with a fluorescence microscope (ThermoFisher EVOS FL Auto Cell Imaging System).

### Isolation of mammary epithelial cells and colony-forming assays

Mammary epithelial cells (MECs) were isolated from mammary glands of wildtype and transgenic mice at pregnancy day 11.5 and lactation day 2 using the EasySep™ Kit (STEMCELL Technologies) according to the manufacturer’s instruction. In brief, mammary glands #3 and #4 (without lymph nodes) were collected and minced with disposable scalpels in 10 cm culture dishes. 5 mL of digestion buffer (DMEM medium containing 1% Pen strep, 10% FBS, 10% gentle collagenase) was added and incubated in a 37 °C hybridization oven for 15 hrs with gentle rotation until no clumps remained. Digested tissues were pelleted by centrifugation at 1000 rpm for 8 min. Accutase (Innovative Cell Technologies, San Diego, CA) was used to dissociate the organoids to obtain mostly single cells. Then the mouse epithelial cell enrichment EasySep™ kit was used to isolate the epithelial cells by sequentially adding the cocktail of Biotinylated antibodies, EasySep™ Biotin Selection cocktail, EasySep™ Magnetic Nanoparticles, and then cells were incubated with the magnet. The enriched epithelial cells were counted and 20,000 cells were seeded in 60 mm plates, cultured for 10 days in EpiCult-B Mouse Medium (StemCell Technologies) supplemented with rhEGF (02633), rhbFGF (02634), Heparin (07980, StemCell Technologies). Then the cells were fixed in 10% formalin for 10 min and stained with crystal violet solution. HC11 cells were seeded in 6-well plates and transfected with pCMV6 vector and pEGFP-FOXC1 plasmid. After 6 days of culture, colonies were stained. The images were taken using an EVOS FL Auto fluorescence microscope (Life Technologies, Carlsbad, CA).

### Chromatin Immunoprecipitation

Approximately 5 × 10^6^ cells of HC11 cells were subjected to ChIP assays, which were conducted using the EZ-ChIP Chromatin Immunoprecipitation Kit (EMD Millipore) according to the manufacturer’s manual. Five micrograms of antibodies were used for each pull-down assay. Immunoprecipitated DNA fragments were purified with the QIAquick Spin Kit (Qiagen) and analyzed by PCR. The primers are *ELF5* forward (5′-TGCCTAGTGATACAGGGTCTCAT-3′) and *ELF5* reverse (5′-CCAACACTCAGGCGGCAGAT-3′). Anti-RNA polymerase II antibody and primers are provided in the ChIP Kit as positive controls.

### RNA-Seq Analysis

RNA was extract form the tissue slides using the RecoverAll™ Total Nucleic Acid Isolation Kit (ThermoFisher). Libraries for RNA-Seq were prepared with Kapa Hyper Prep Kits with RiboErase. The workflow consists of rRNA depletion, cDNA generation, and end repair to generate blunt ends, A-tailing, adaptor ligation and PCR amplification. Different adaptors were used for multiplexing samples in one lane. Sequencing was performed on Illumina Hiseq. 3000 for a single read 50 run. Data quality check was done on Illumina SAV. Demultiplexing was performed with Illumina Bcl2fastq2 v 2.17 program. Tophat2 (2.0.7)^43^ was applied to align sequencing reads to the reference mouse genome (GRCm38/mm10). The relative gene expression RPKM and read counts were estimated using SAMMate (2.7.4)^44^ and Ensembl database (Mus Musculus.GRCm38.82). Protein coding genes with at least 2 RPKM on average in either condition were used to perform the differential gene expression analysis (MMTV-FOXC1 transgenic vs. wildtype mice) using DESeq. 2^45^. The *p*-values of multiple tests were adjusted using Benjamini-Hochberg’s method^46^ and the significant level was designed as FDR < 0.01 and |log2 FC| > 2. RNA-Seq data in this study is available in the NCBI GEO database under the accession number GSE104443. Pathway enrichment analysis of differentially expressed genes was performed using DAVID^47^.

## Electronic supplementary material

Supplementary information
