# Supplementary material for: Inhibition of lobuloalveolar development by FOXC1 overexpression in the mouse mammary gland
Source: Sci Rep. 2017 Oct 25;7:14017. doi: 10.1038/s41598-017-14342-8 (PMC5656618; doi:10.1038/s41598-017-14342-8)
Supplement: Supplementary file 1 — Supplementary information [file 41598_2017_14342_MOESM1_ESM.pdf]

Supplementary Information

Title: Inhibition of lobuloalveolar development by FOXC1 overexpression in the mouse mammary gland

Authors: Bowen Gao[1], Ying Qu[1], Bingchen Han[1], Yoshiko Nagaoka[2], Makoto Katsumata[2], Nan Deng[3], Shikha Bose[4], Liting Jin[1,5], Armando E. Giuliano[1], Xiaojiang Cui[1]

Supplementary Figure Legends

Figure S1. Generation of MMTV-FOXC1 transgenic mice.

(a) Illustration of the MMTV-FOXC1 expression construct. (b) Identification of founder mice using PCR analysis of mouse tail genomic DNA. (c) Immunohistochemistry analysis for tissue specificity of FOXC1 transgene expression in heart, liver, spleen, lung, kidney, intestine, pancreas, ovary, brain, and stomach tissues isolated from wildtype and transgenic mice.

Figure S2. Effect of FOXC1 overexpression on Ki67+ mammary cells.

Immunohistochemistry of Ki67 in mammary gland tissues collected at different development stages: virgin week 10, pregnancy day 11.5, lactation day 2, and involution day 7 from wildtype and transgenic mice. Magnification, x60. Comparison of percentage of Ki67+ cells at pregnancy day 11.5 and lactation day 2 from wildtype and transgenic mice is graphed. The bar graph represents mean ± SD (n = 5).**, $p < 0.01$.

Figure S3. Effect of FOXC1 overexpression on PRLR, Elf5, and p-Stat5 levels.

Total RNA was isolated from the mammary tissue of wildtype and transgenic mice at lactation day 2. Real-time RT-PCR was performed. (a) Comparison of PRLR expression. (b) Comparison of Elf5 expression. The bar graph represents mean ± SD (n = 3). **, $p < 0.01$. (c) Immunohistochemistry of Elf5 in the wildtype and transgenic mice at virgin week 10 and early pregnancy day 11.5. (d) Recruitment of FOXC1 to the Elf5 promoter region containing a potential bind site (at -1360 bp from the transcription start site) was tested by ChIP-PCR analysis. The RNA Polymerase II (Pol II) protein and associated PCR primers were used as positive controls. (e) Comparison of p-Stat5 levels in vector- and FOXC1-transfected HC11 cells by immunoblotting. Cells were treated with vehicle or prolactin (1 μg/ml) for 30 min before cell lysates were harvested. For the full-length image, see Figure S5.

Figure S4. Colony-forming assays with mouse epithelial cells.

(a) Colony-forming assays with mouse epithelial cells from wildtype and transgenic mice at pregnancy day 11.5. The bar graph indicates mean ± SD (n = 3). Same results of virgin week 10 were also oberseved (data not shown). (b) Colony-forming assays with vector- and FOXC1-transfected HC11 mouse epithelial cells. The bar graph indicates mean ± SD (n = 3). The images were obtained with magnification x10. ***, $p < 0.001$.

Figure S5. Full-length western blot images.

(a) Full-length western blot image for Figure 1a. (b) Full-length western blot image for Figure 3c. (c) Full-length western blot image for Figure S3e.

Figure S1

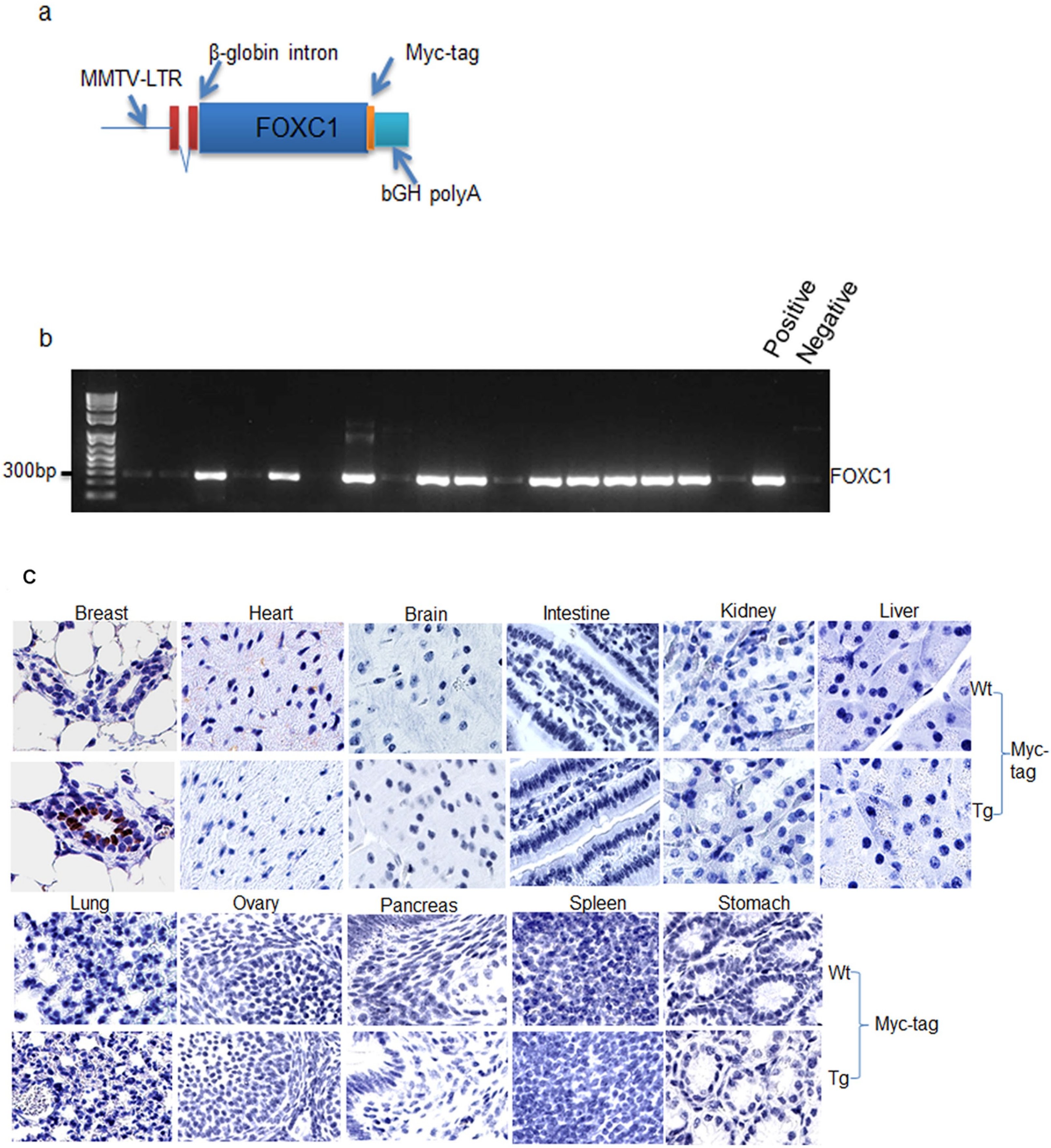

a
β-globin intron
Myc-tag
MMTV-LTR
FOXC1
bGH polyA
b
Positive
Negative
300bp
FOXC1
c
Breast
Heart
Brain
Intestine
Kidney
Liver
Wt
Myc-tag
Tg
Lung
Ovary
Pancreas
Spleen
Stomach
Wt
Myc-tag
Tg

Figure S2

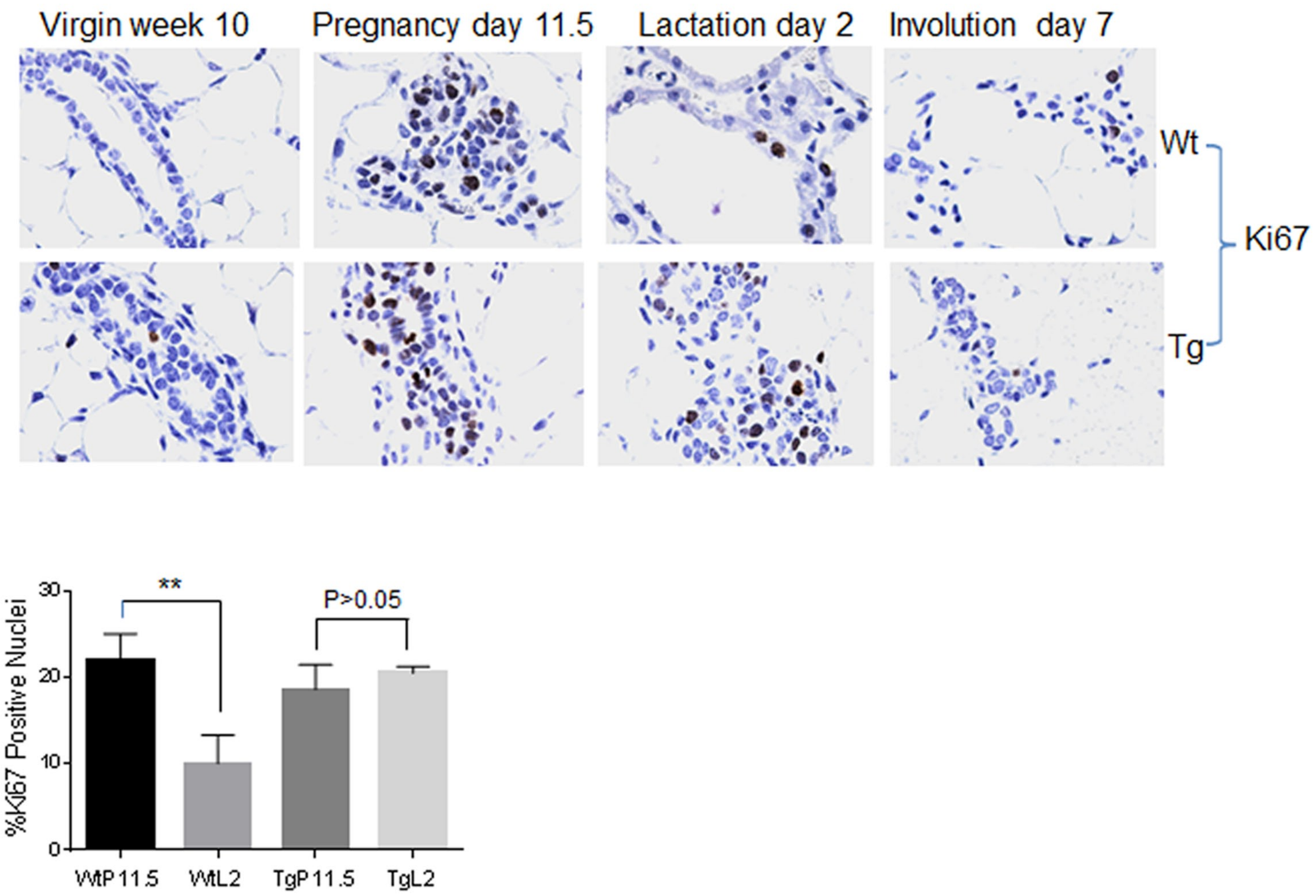
Virgin week 10
Pregnancy day 11.5
Lactation day 2
Involution day 7
Wt
Tg
Ki67
%Ki67 Positive Nuclei
30
20
10
0
**
P>0.05
WtP11.5
WtL2
TgP11.5
TgL2

Figure S3

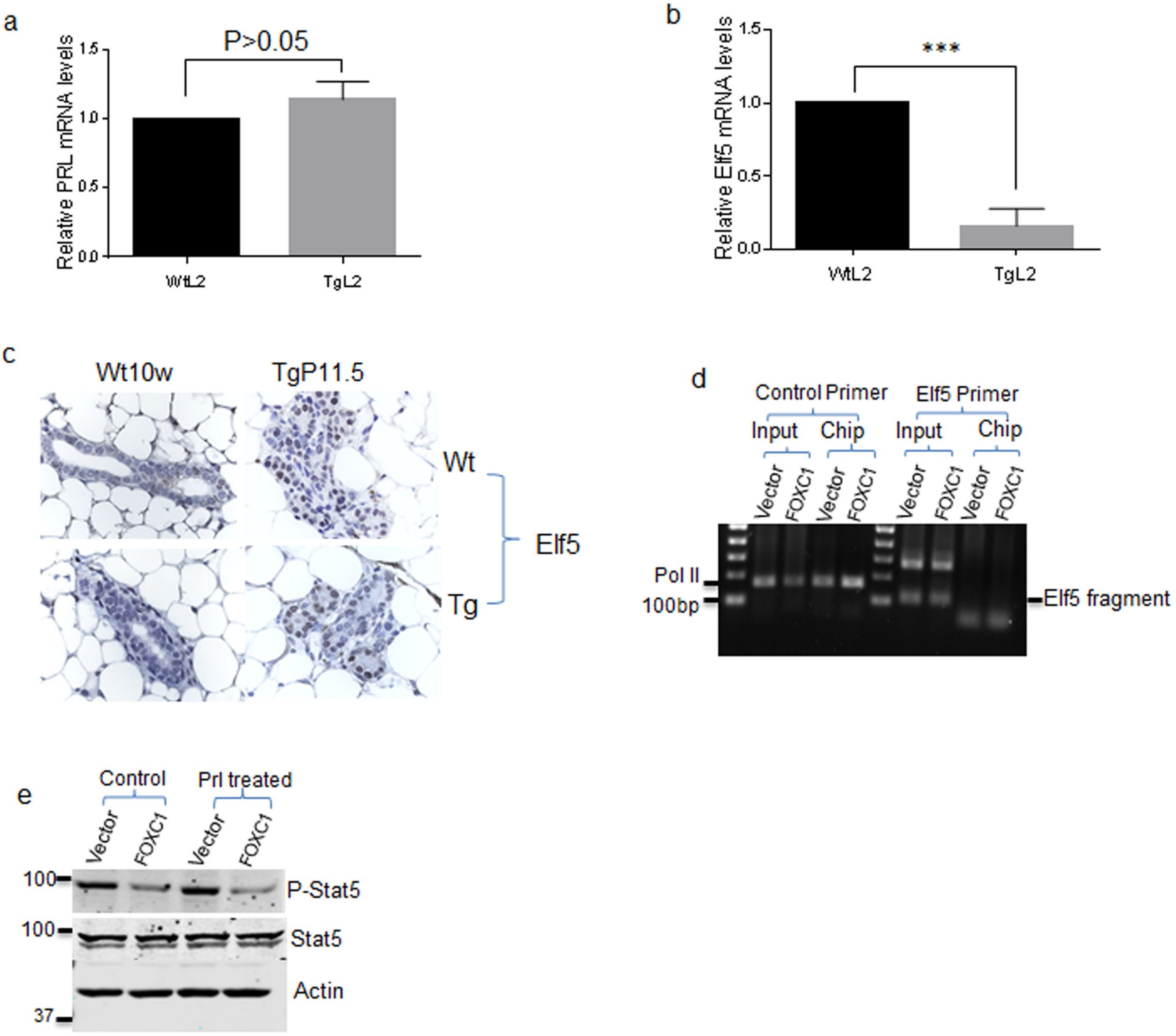
a
P>0.05
Relative PRL mRNA levels
1.5
1.0
0.5
0.0
WtL2
TgL2
b
***
Relative Elf5 mRNA levels
1.5
1.0
0.5
0.0
WtL2
TgL2
c
Wt10w
TgP11.5
Wt
Tg
Elf5
d
Control Primer
Elf5 Primer
Input
Chip
Input
Chip
Vector
FOXC1
Vector
FOXC1
Vector
FOXC1
Vector
FOXC1
Pol II
100bp
Elf5 fragment
e
Control
Prl treated
Vector
FOXC1
Vector
FOXC1
100
P-Stat5
100
Stat5
Actin
37

Figure S4

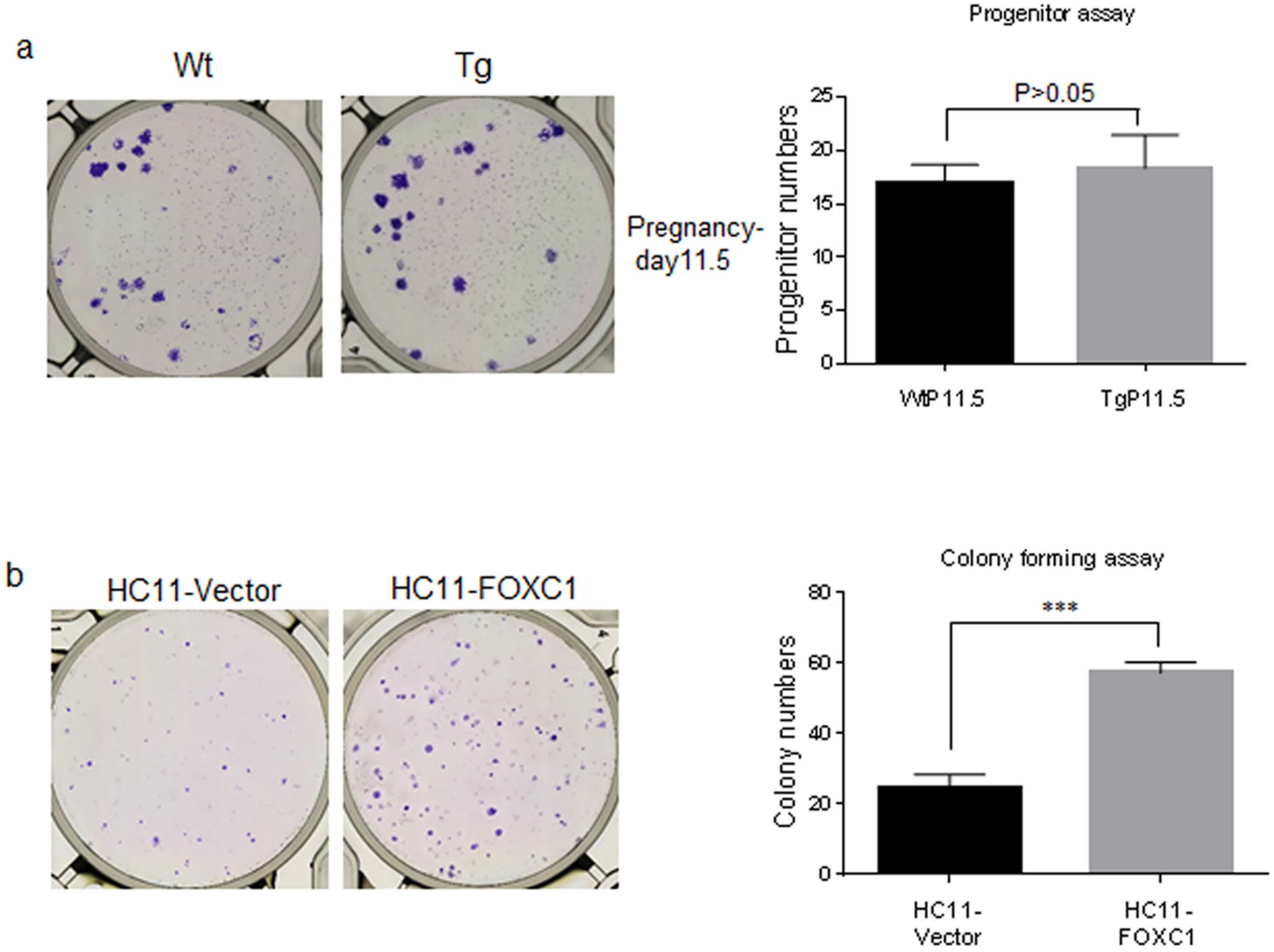
a
Wt
Tg
Pregnancy-
day11.5
Progenitor assay
Progenitor numbers
25
20
15
10
5
0
P>0.05
WtP11.5
TgP11.5
b
HC11-Vector
HC11-FOXC1
Colony forming assay
Colony numbers
80
60
40
20
0
***
HC11-
Vector
HC11-
FOXC1

Figure S5

Figure 1a, full-length image

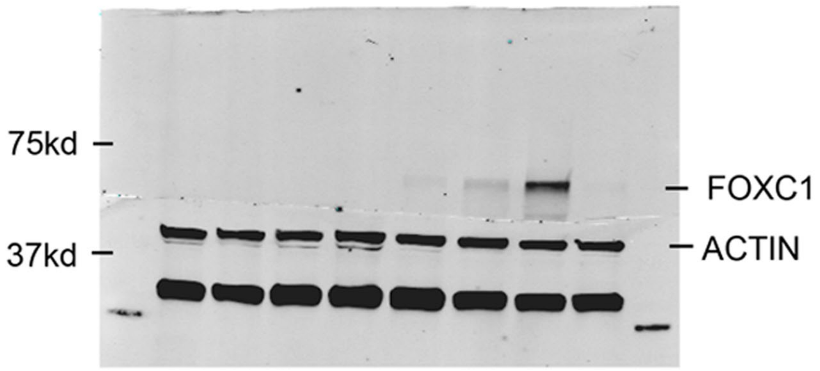

Figure 3c, full-length image

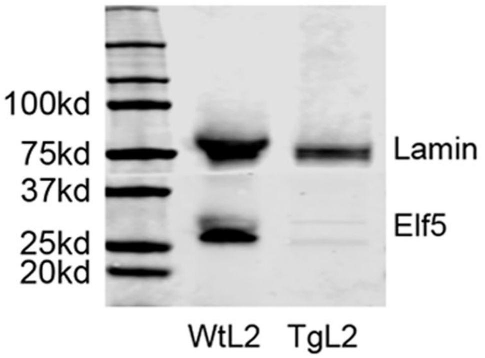

Figure S.3e, full length image

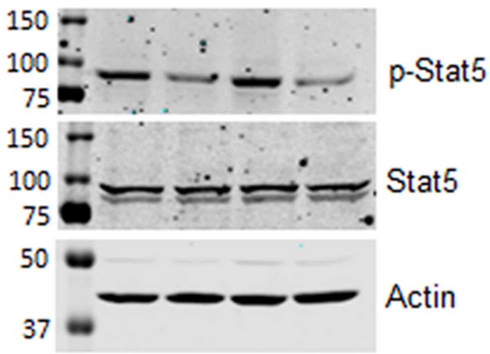

Supplementary Table S1. Top differentially expressed genes in the mammary glands between wildtype and transgenic mice.

RNA Seq was performed using total RNA isolated from the mammary tissue of wildtype (Wt) and transgenic (Tg) mice at day 2 of the lactation stage, followed by gene expression analysis (for details, see Materials and Methods). Top 200 up- or down-regulated genes (Tg/Wt) are listed.

Top Differentially Regulated Genes (Tg/Wt)

GeneID	GeneName	Log2FoldChange	FoldChange	Pvalue	FDR	Filter
ENSMUSG00000006269	Atp6v1b1	10.98262904	2023.488641	3.07E-05	5.42E-05	1
ENSMUSG00000042474	Fcmr	10.39396911	1345.539575	8.03E-05	0.000136368	1
ENSMUSG00000027368	Dusp2	10.00440619	1027.132222	0.000147579	0.000244252	1
ENSMUSG00000043635	Adamts3	10.0028433	1026.020113	0.000147786	0.000244563	1
ENSMUSG00000047861	Foxi1	9.920269646	968.944113	0.000167803	0.000276481	1
ENSMUSG00000073406	H2-Bl	9.878448248	941.2591985	0.000190496	0.000312327	1
ENSMUSG00000040276	Pacsin1	9.733665028	851.3833272	0.000222706	0.000363681	1
ENSMUSG00000022416	Cacna1i	9.720156931	843.4489438	0.000227819	0.000371662	1
ENSMUSG00000018924	Alox15	9.712302336	838.8693566	0.000229859	0.000374663	1
ENSMUSG00000055546	Timd4	9.698497614	830.8807448	0.000234672	0.000382223	1
ENSMUSG00000059994	Fcrl1	9.641912066	798.9226748	0.000255599	0.000415277	1
ENSMUSG00000026009	Icos	9.550414267	749.8271567	0.000292353	0.000472767	1
ENSMUSG00000071715	Ncf4	9.407216569	678.9759053	0.000365891	0.000586624	1
ENSMUSG00000031710	Ucp1	9.227819144	599.5844442	1.63E-10	4.05E-10	1
ENSMUSG00000051397	Tacstd2	9.17654334	578.6484603	0.000503073	0.000796729	1
ENSMUSG00000034959	5031414D18Rik	9.13902447	563.7940609	0.000530351	0.000837302	1
ENSMUSG00000063011	Msln	9.106388701	551.1834277	0.000561479	0.000884636	1
ENSMUSG00000020607	Fam84a	9.082375983	542.0852655	0.000576363	0.000906888	1
ENSMUSG00000038421	Fcrla	9.079555119	541.0263758	0.000586767	0.00092239	1
ENSMUSG00000020732	Rab37	9.070371127	537.5932177	0.000589693	0.00092653	1
ENSMUSG00000022382	Wnt7b	9.070166078	537.5168155	0.000586521	0.000922208	1
ENSMUSG00000027656	Wisp2	9.064096042	535.260003	0.000589909	0.000926647	1
ENSMUSG00000022876	Samsn1	9.0235646	520.4315446	0.000624803	0.000978296	1
ENSMUSG00000068758	Il3ra	8.985078334	506.7317214	0.000659254	0.00102978	1
ENSMUSG00000029671	Wnt16	8.968746629	501.0277252	0.000674466	0.001052414	1
ENSMUSG00000092130	D030025P21Rik	8.965429283	499.8769809	0.000679858	0.001059946	1
ENSMUSG00000063975	Slco1a5	8.932151888	488.4787147	0.000710429	0.001105111	1
ENSMUSG00000024334	H2-Oa	8.827336971	454.248195	0.00083556	0.001290273	1
ENSMUSG00000027297	Ltk	8.764315961	434.8324995	0.000895241	0.001378377	1
ENSMUSG00000094065	Gm21541	8.72199798	422.2629855	0.000949681	0.00145826	1
ENSMUSG00000073878	Gm13304	8.702472617	416.5865981	0.000975967	0.001496871	1
ENSMUSG00000022435	Upk3a	8.678405397	409.6946938	0.001012842	0.001550707	1
ENSMUSG00000038754	Elovl3	8.650223215	401.7692153	0.001052961	0.001608752	1
ENSMUSG00000026322	Htr4	8.604334344	389.1909475	0.001118779	0.001704942	1
ENSMUSG00000041673	Lrrc18	8.587293074	384.6208265	0.00113968	0.001735181	1
ENSMUSG00000018907	Alox12e	8.576627955	381.7880018	4.51E-17	1.51E-16	1
ENSMUSG00000004612	Nkg7	8.539363625	372.0528151	0.0012172	0.001848273	1
ENSMUSG00000036596	Cpz	8.498812683	361.740842	0.001300008	0.001969227	1
ENSMUSG00000038599	Capn8	8.44327093	348.0789911	0.001386802	0.002094171	1
ENSMUSG00000061414	Cracr2a	8.370564246	330.9717443	0.001521677	0.002286262	1
ENSMUSG00000030724	Cd19	8.365875472	329.8978287	6.89E-09	1.56E-08	1
ENSMUSG00000042451	Mybph	8.355582636	327.552565	0.001552257	0.002329602	1
ENSMUSG00000049086	Bmyc	8.33534062	322.9888654	0.001597366	0.002392037	1
ENSMUSG00000036526	Card11	8.297012891	314.5210774	9.20E-09	2.07E-08	1
ENSMUSG00000048450	Msx1	8.288537827	312.6788502	0.001711379	0.002554023	1
ENSMUSG00000036813	Entpd8	8.28339843	311.5669576	0.001707268	0.002548759	1
ENSMUSG00000074228	Gm10645	8.207397024	295.5783978	0.001894738	0.002818366	1
ENSMUSG00000003484	Cyp4f18	8.185095667	291.044444	0.001948299	0.002893771	1
ENSMUSG00000074625	Arhgap40	8.139087002	281.9092492	0.002061624	0.003054137	1
ENSMUSG00000066607	6030419C18Rik	8.138816896	281.8564742	0.002104733	0.00311378	1
ENSMUSG00000039760	Il22ra2	8.133155963	280.752676	0.002075133	0.003072762	1
ENSMUSG00000026011	Ctla4	8.10955583	276.1973812	0.002146291	0.003172041	1
ENSMUSG00000039481	Nrtn	8.097433994	273.886433	0.002184933	0.003225881	1
ENSMUSG00000090863	A530084C06Rik	8.054916695	265.9325744	0.002299415	0.003384239	1

ENSMUSG00000028871	Rspo1	8.054886312	265.9269739	0.002294925	0.00337801	1
ENSMUSG00000021013	Ttc8	8.029958789	261.3716379	0.002376078	0.00349276	1
ENSMUSG00000067341	H2-Eb2	7.978575261	252.2263622	0.002533666	0.003716082	1
ENSMUSG00000039476	Prrx2	7.904936264	239.6751067	0.002779841	0.004059893	1
ENSMUSG00000014030	Pax5	7.883742253	236.1798793	1.18E-14	3.57E-14	1
ENSMUSG00000022057	Adamdec1	7.870147871	233.9648305	0.002901098	0.004228981	1
ENSMUSG00000079014	Serpina3i	7.775595298	219.1227244	0.003299401	0.00477985	1
ENSMUSG00000024670	Cd6	7.773445231	218.7964061	7.42E-08	1.57E-07	1
ENSMUSG00000041750	Cd1d2	7.768068768	217.9825394	0.003296057	0.004775533	1
ENSMUSG00000035930	Chst4	7.706323793	208.8500665	0.003572198	0.00515626	1
ENSMUSG00000037944	Ccr7	7.674633299	204.3124486	1.10E-07	2.32E-07	1
ENSMUSG00000054200	Ffar4	7.650020535	200.8563879	0.0038216	0.005491483	1
ENSMUSG00000024535	Snx24	7.64198994	199.7414501	0.003863067	0.00554803	1
ENSMUSG00000050742	Olfr164	7.616856484	196.2918528	0.003980159	0.005708071	1
ENSMUSG00000043644	0610009L18Rik	7.600134231	194.0297726	0.004058369	0.005818326	1
ENSMUSG00000023755	Rhebl1	7.591520524	192.8747563	0.00414033	0.005931293	1
ENSMUSG00000043219	Hoxa6	7.512578065	182.6044439	0.004516293	0.006444548	1
ENSMUSG00000032172	Olfm2	7.485280303	179.1818009	0.004668187	0.00665261	1
ENSMUSG00000094947	Gm3944	7.466667539	176.8849556	0.004791072	0.006818844	1
ENSMUSG00000043687	1190005I06Rik	7.419639259	171.2119107	0.005085445	0.007211985	1
ENSMUSG00000094686	Ccl21a	7.370820586	165.5152785	3.39E-07	6.92E-07	1
ENSMUSG00000064109	Hcst	7.309703175	158.649941	0.005807978	0.008186209	1
ENSMUSG00000097187	Gm19426	7.235170884	150.6619068	0.006297514	0.008834434	1
ENSMUSG00000003379	Cd79a	7.214433853	148.5118101	5.49E-18	1.91E-17	1
ENSMUSG00000028445	Enho	7.133177933	140.3784756	0.007087163	0.009900895	1
ENSMUSG00000047501	Cldn4	7.110437691	138.1831305	3.52E-12	9.51E-12	1
ENSMUSG00000015396	Cd83	6.744107138	107.1959907	3.14E-06	6.00E-06	1
ENSMUSG00000045322	Tlr9	6.732254881	106.3189448	3.32E-06	6.34E-06	1
ENSMUSG00000001436	Slc19a1	6.575316186	95.3602548	5.62E-06	1.05E-05	1
ENSMUSG00000070000	Fcho1	6.512955908	91.32613717	2.00E-10	4.94E-10	1
ENSMUSG00000014453	Blk	6.472585863	88.80603789	7.76E-06	1.44E-05	1
ENSMUSG00000030905	Crym	6.44368273	87.04459002	8.64E-06	1.60E-05	1
ENSMUSG00000079563	Pglyrp2	6.431002093	86.28285999	8.97E-06	1.66E-05	1
ENSMUSG00000037337	Map4k1	6.381707143	83.38448938	4.71E-10	1.14E-09	1
ENSMUSG00000040592	Cd79b	6.334160445	80.68118928	6.16E-10	1.48E-09	1
ENSMUSG00000044217	Aqp5	6.326298439	80.24271076	1.27E-05	2.32E-05	1
ENSMUSG00000006777	Krt23	6.317073253	79.73124285	1.30E-05	2.37E-05	1
ENSMUSG00000013338	Fer1l4	6.30565402	79.10264442	1.33E-05	2.43E-05	1
ENSMUSG00000024669	Cd5	6.28378111	77.91240319	8.69E-10	2.07E-09	1
ENSMUSG00000047730	Fcgbp	6.265772049	76.94587325	3.73E-160	8.58E-159	1
ENSMUSG00000046782	4921506M07Rik	6.263901054	76.84614875	1.65E-05	2.98E-05	1
ENSMUSG00000035896	Rnase1	6.195892568	73.30768634	1.90E-05	3.41E-05	1
ENSMUSG00000064246	Chil1	6.194724664	73.24836564	1.19E-17	4.07E-17	1
ENSMUSG00000000486	Sept1	6.172438376	72.1255433	1.79E-21	7.00E-21	1
ENSMUSG00000045165	AI467606	6.159490681	71.48113717	1.91E-09	4.48E-09	1
ENSMUSG00000034833	Tespa1	5.985562595	63.36273019	3.66E-05	6.42E-05	1
ENSMUSG00000027239	Mdk	5.971098608	62.73065007	5.71E-09	1.30E-08	1
ENSMUSG00000024598	Fbn2	5.943052242	61.52292721	5.44E-20	2.02E-19	1
ENSMUSG00000026581	Sell	5.902217652	59.80597216	3.01E-34	1.66E-33	1
ENSMUSG00000023078	Cxcl13	5.797313371	55.61157806	1.59E-08	3.52E-08	1
ENSMUSG00000013707	Tnfaip8l2	5.775944984	54.79396066	6.83E-05	0.000116791	1
ENSMUSG00000092021	Gbp11	5.772887584	54.67796269	7.47E-05	0.00012713	1
ENSMUSG00000031933	Izumo1r	5.766006598	54.41779512	7.02E-05	0.000119995	1
ENSMUSG00000035451	Foxa1	5.732132121	53.15494921	2.89E-15	8.97E-15	1
ENSMUSG00000041538	H2-Ob	5.712247169	52.42732999	1.38E-18	4.90E-18	1
ENSMUSG00000037922	Bank1	5.709784395	52.33790947	5.40E-22	2.15E-21	1
ENSMUSG00000052013	Btla	5.687893322	51.54974272	4.55E-15	1.40E-14	1

ENSMUSG00000039883	Lrrc17	5.636804373	49.75619897	0.000102721	0.000172733	1
ENSMUSG00000040740	Slc25a34	5.607228992	48.74657638	0.000111902	0.000187355	1
ENSMUSG00000030798	Cd37	5.594893904	48.33156831	6.56E-44	4.41E-43	1
ENSMUSG00000040247	Tbc1d10c	5.585820037	48.02853962	2.37E-24	1.01E-23	1
ENSMUSG00000093668	Pou5f2	5.577079384	47.7384356	0.000122738	0.000204845	1
ENSMUSG00000033826	Dnah8	5.522315178	45.96026395	4.68E-49	3.42E-48	1
ENSMUSG00000031779	Ccl22	5.492902938	45.03275817	1.96E-20	7.39E-20	1
ENSMUSG00000025163	Cd7	5.486832785	44.84368049	0.000157403	0.000259921	1
ENSMUSG00000034634	Ly6d	5.478667226	44.59058514	6.37E-11	1.62E-10	1
ENSMUSG00000045502	Hcar2	5.453833848	43.8296073	0.000182967	0.000300508	1
ENSMUSG00000069267	Hist1h3b	5.41610324	42.69819681	0.000196453	0.000321892	1
ENSMUSG00000026390	Marco	5.323920108	40.05526849	0.00024909	0.000405003	1
ENSMUSG00000030707	Coro1a	5.285222619	38.99514523	2.00E-82	2.40E-81	1
ENSMUSG00000038415	Foxq1	5.247153507	37.97961845	3.35E-07	6.83E-07	1
ENSMUSG00000051506	Wdfy4	5.246746123	37.96889539	1.42E-88	1.85E-87	1
ENSMUSG00000050357	Rltpr	5.227862939	37.47516536	6.54E-13	1.83E-12	1
ENSMUSG00000024402	Lta	5.191045765	36.53090943	0.000359864	0.000577668	1
ENSMUSG00000000673	Haao	5.160766197	35.77218154	0.000388788	0.000622345	1
ENSMUSG00000050600	Zfp831	5.15519926	35.63441315	4.83E-29	2.35E-28	1
ENSMUSG00000037849	Gm4955	5.140385427	35.27038521	0.000407627	0.000651627	1
ENSMUSG00000074766	Ism1	5.129998364	35.01735873	0.000422841	0.000674633	1
ENSMUSG00000037280	Galnt6	5.124120576	34.8749822	1.26E-20	4.81E-20	1
ENSMUSG00000030244	Gys2	5.11958713	34.76556499	6.60E-07	1.32E-06	1
ENSMUSG00000091938	Gm2564	5.116930652	34.701609	6.63E-07	1.33E-06	1
ENSMUSG00000051457	Spn	5.116018651	34.67967927	9.93E-37	5.78E-36	1
ENSMUSG00000043931	Gimap7	5.090682126	34.07595362	8.09E-07	1.61E-06	1
ENSMUSG00000035697	Hmha1	5.03523673	32.79119856	2.55E-147	5.48E-146	1
ENSMUSG00000022696	Sidt1	5.003454307	32.07671077	1.77E-45	1.22E-44	1
ENSMUSG00000059674	Cdh24	4.994171112	31.87097196	0.000608028	0.000953054	1
ENSMUSG00000024399	Ltb	4.994087626	31.8691277	2.07E-27	9.58E-27	1
ENSMUSG00000042345	Ubash3a	4.969089421	31.32167412	3.61E-09	8.31E-09	1
ENSMUSG00000025473	Adam8	4.965312747	31.23978777	3.58E-09	8.24E-09	1
ENSMUSG00000094958	3110021N24Rik	4.953625215	30.98773098	0.00066706	0.001041354	1
ENSMUSG00000031725	Ces1f	4.943037497	30.76114921	3.45E-14	1.02E-13	1
ENSMUSG00000030742	Lat	4.934166083	30.57257328	4.15E-19	1.49E-18	1
ENSMUSG00000100621	Gm28374	4.929913835	30.48259532	0.000713016	0.001108873	1
ENSMUSG00000000682	Cd52	4.922789324	30.33243312	1.29E-26	5.86E-26	1
ENSMUSG00000005465	Il27ra	4.920022431	30.27431539	4.45E-24	1.88E-23	1
ENSMUSG00000034041	Lyl1	4.899859772	29.8541538	1.78E-11	4.64E-11	1
ENSMUSG00000066366	Serpina1a	4.899791341	29.85273778	0.000769804	0.001192523	1
ENSMUSG00000020728	Cep112	4.89373452	29.72767068	2.09E-06	4.03E-06	1
ENSMUSG00000041481	Serpina3g	4.872217238	29.28758311	2.27E-06	4.37E-06	1
ENSMUSG00000092277	Gm19684	4.869175544	29.22589992	0.000839066	0.001295383	1
ENSMUSG00000032271	Nnmt	4.85026127	28.84523817	2.57E-06	4.93E-06	1
ENSMUSG00000000142	Axin2	4.846140108	28.76295724	2.42E-23	9.98E-23	1
ENSMUSG00000079015	Serpina1c	4.844049774	28.72131251	0.000893906	0.001376806	1
ENSMUSG00000031722	Hp	4.837641486	28.59401861	8.64E-26	3.83E-25	1
ENSMUSG00000027875	Hmgcs2	4.828879905	28.42089152	7.36E-21	2.82E-20	1
ENSMUSG00000023828	Slc22a3	4.805493226	27.96389129	1.17E-08	2.62E-08	1
ENSMUSG00000018168	Ikzf3	4.804193661	27.93871303	8.13E-61	7.16E-60	1
ENSMUSG00000025017	Pik3ap1	4.800612945	27.86945617	1.41E-20	5.36E-20	1
ENSMUSG00000020424	Gatsl3	4.792279773	27.708943	0.001054592	0.001610868	1
ENSMUSG00000010660	Plcd1	4.78357487	27.54225673	1.48E-08	3.29E-08	1
ENSMUSG00000026413	Pkp1	4.762847415	27.14938128	2.53E-41	1.62E-40	1
ENSMUSG00000020926	Adam11	4.761909454	27.13173598	3.48E-13	9.87E-13	1
ENSMUSG00000023906	Cldn6	4.745693781	26.82848682	0.001151952	0.001753459	1
ENSMUSG00000029061	Mmp23	4.745686179	26.82834545	4.35E-06	8.24E-06	1

ENSMUSG00000038775	Vill	4.729465397	26.52839332	4.07E-13	1.15E-12	1
ENSMUSG00000040938	Slc16a11	4.718571255	26.32882539	5.31E-06	9.99E-06	1
ENSMUSG00000024331	Dsc2	4.71672448	26.29514377	9.98E-11	2.51E-10	1
ENSMUSG00000024176	Sox8	4.708011235	26.13681126	5.20E-06	9.78E-06	1
ENSMUSG00000078141	Gm2399	4.697468121	25.94650151	0.001340213	0.002027319	1
ENSMUSG00000032093	Cd3e	4.685064636	25.72438419	3.22E-42	2.09E-41	1
ENSMUSG00000054474	Thnsl2	4.669243852	25.44382832	0.001372956	0.002074457	1
ENSMUSG00000030830	Itgal	4.636495388	24.87277187	1.45E-54	1.16E-53	1
ENSMUSG00000054320	Lrrc36	4.61844564	24.56352389	0.001552299	0.002329602	1
ENSMUSG00000052087	Rgs14	4.609125673	24.4053523	2.71E-10	6.65E-10	1
ENSMUSG00000044734	Serpinb1a	4.603525633	24.31080285	8.62E-06	1.59E-05	1
ENSMUSG00000052142	Rasal3	4.592925935	24.13284226	4.07E-64	3.76E-63	1
ENSMUSG00000028159	Dapp1	4.584139492	23.98631271	1.22E-16	3.99E-16	1
ENSMUSG00000071005	Ccl19	4.580935057	23.93309475	2.27E-12	6.20E-12	1
ENSMUSG00000047959	Kcna3	4.571412201	23.77563883	3.84E-10	9.34E-10	1
ENSMUSG00000068227	Il2rb	4.568315185	23.7246547	1.24E-20	4.71E-20	1
ENSMUSG00000034059	Ypel4	4.561844126	23.61847854	9.86E-06	1.82E-05	1
ENSMUSG00000041515	Irf8	4.555374103	23.51279442	1.65E-62	1.48E-61	1
ENSMUSG00000000244	Tspan32	4.539120843	23.24938812	2.00E-16	6.50E-16	1
ENSMUSG00000064023	Klk8	4.534669853	23.17776986	0.00191649	0.002848783	1
ENSMUSG00000051998	Lax1	4.529981694	23.10257398	4.38E-12	1.18E-11	1
ENSMUSG00000030091	Nup210	4.515639715	22.87404671	4.08E-68	4.04E-67	1
ENSMUSG00000044628	Rnf208	4.510841586	22.79809836	0.00209008	0.0030928	1
ENSMUSG00000055805	Fmnl1	4.507329335	22.74266373	4.08E-66	3.91E-65	1
ENSMUSG00000000409	Lck	4.50409899	22.6917975	7.00E-43	4.61E-42	1
ENSMUSG00000031304	Il2rg	4.498722715	22.60739276	4.06E-49	2.97E-48	1
ENSMUSG00000092463	Gm20489	4.496482584	22.57231658	6.67E-12	1.78E-11	1
ENSMUSG00000047003	Zfp41	4.494910319	22.54773043	1.35E-05	2.46E-05	1
ENSMUSG00000015355	Cd48	4.48681044	22.42149296	1.46E-05	2.64E-05	1
ENSMUSG00000030170	Wnt5b	4.480812911	22.32847648	1.04E-09	2.47E-09	1
ENSMUSG00000048138	Dmrt2	4.456764123	21.9593603	1.29E-07	2.71E-07	1
ENSMUSG00000020437	Myo1g	4.447690204	21.82167899	7.58E-52	5.81E-51	1
ENSMUSG00000034384	Barhl2	-3.482579508	0.089462104	1.95E-76	2.18E-75	1
ENSMUSG00000022790	Igsf11	-3.484184196	0.089362652	5.70E-102	8.41E-101	1
ENSMUSG00000030031	Kbtbd8	-3.487947799	0.089129832	2.21E-73	2.36E-72	1
ENSMUSG00000016526	Dyrk3	-3.4879935	0.089127009	7.00E-45	4.80E-44	1
ENSMUSG00000027995	Tlr2	-3.505700769	0.088039773	4.03E-182	1.06E-180	1
ENSMUSG00000025289	Prdx4	-3.513027485	0.087593797	3.99E-259	1.50E-257	1
ENSMUSG00000041617	Ccdc74a	-3.513260983	0.087579621	7.62E-50	5.65E-49	1
ENSMUSG00000000594	Gm2a	-3.514168376	0.087524554	0	0	1
ENSMUSG00000000792	Slc5a5	-3.526335662	0.086789501	1.70E-88	2.20E-87	1
ENSMUSG00000030659	Nucb2	-3.532460961	0.086421797	0	0	1
ENSMUSG00000000983	Wfdc18	-3.533896254	0.086335861	0	0	1
ENSMUSG00000024530	Slmo1	-3.540228046	0.085957775	6.97E-138	1.41E-136	1
ENSMUSG00000024665	Fads2	-3.545457126	0.085646783	0	0	1
ENSMUSG00000040601	Nlrp4a	-3.551488715	0.085289461	4.46E-51	3.37E-50	1
ENSMUSG00000039168	Dap	-3.552207227	0.085246994	0	0	1
ENSMUSG00000035653	Lrfn5	-3.555196593	0.085070539	1.09E-162	2.55E-161	1
ENSMUSG00000043557	Mdga1	-3.561059397	0.084725532	7.27E-139	1.48E-137	1
ENSMUSG00000096024	Gm17174	-3.570859538	0.084151947	4.61E-75	5.06E-74	1
ENSMUSG00000024066	Xdh	-3.579408854	0.083654743	0	0	1
ENSMUSG00000030110	Ret	-3.591580402	0.082951944	2.17E-102	3.22E-101	1
ENSMUSG00000032313	AI118078	-3.624759273	0.081065998	2.61E-42	1.70E-41	1
ENSMUSG00000043487	Acot6	-3.626335557	0.080977474	2.74E-163	6.46E-162	1
ENSMUSG00000044165	Bcl2l15	-3.63960437	0.080236119	1.90E-63	1.74E-62	1
ENSMUSG00000061013	Mkx	-3.646934714	0.079829472	7.47E-164	1.77E-162	1
ENSMUSG00000003528	Slc25a1	-3.656078446	0.079325117	0	0	1

ENSMUSG00000049307	Fut4	-3.663946388	0.078893684	8.91E-154	1.99E-152	1
ENSMUSG00000032532	Cck	-3.675923367	0.078241435	2.23E-71	2.32E-70	1
ENSMUSG00000050270	Tmem220	-3.679779895	0.078032563	6.24E-186	1.68E-184	1
ENSMUSG00000046352	Gjb2	-3.680988203	0.077967236	0	0	1
ENSMUSG00000006221	Hspb7	-3.69322929	0.077308492	1.04E-197	2.99E-196	1
ENSMUSG00000090223	Pcp4	-3.695199643	0.077202981	4.98E-26	2.22E-25	1
ENSMUSG00000020532	Acaca	-3.697171594	0.077097528	0	0	1
ENSMUSG00000004031	Brinp2	-3.703992355	0.076733887	2.77E-113	4.56E-112	1
ENSMUSG00000022351	Sqle	-3.719538949	0.075911436	0	0	1
ENSMUSG00000017390	Aldoc	-3.722185159	0.075772326	0	0	1
ENSMUSG00000044468	Fam46c	-3.723712199	0.075692166	0	0	1
ENSMUSG00000018846	Pank3	-3.730011432	0.075362392	0	0	1
ENSMUSG00000006345	Ggt1	-3.731396992	0.075290049	1.39E-185	3.72E-184	1
ENSMUSG00000022033	Pbk	-3.743777228	0.074646725	2.91E-37	1.71E-36	1
ENSMUSG00000021591	Glrx	-3.753668669	0.074136681	0	0	1
ENSMUSG00000040434	Gyltl1b	-3.760775407	0.07377238	0	0	1
ENSMUSG00000019913	Sim1	-3.762191466	0.073700005	2.31E-83	2.82E-82	1
ENSMUSG00000002769	Gnmt	-3.766318407	0.073489482	5.16E-28	2.43E-27	1
ENSMUSG00000022756	Slc7a4	-3.772189733	0.07319101	2.70E-153	6.02E-152	1
ENSMUSG00000001542	Ell2	-3.782196768	0.072685088	0	0	1
ENSMUSG00000020638	Cmpk2	-3.79178734	0.072203504	1.25E-244	4.41E-243	1
ENSMUSG00000095139	Pou3f2	-3.798560033	0.071865341	2.66E-71	2.76E-70	1
ENSMUSG00000062609	Kcnj15	-3.799660935	0.071810522	0	0	1
ENSMUSG00000044465	Fam160a2	-3.801487339	0.07171967	0	0	1
ENSMUSG00000015653	Steap2	-3.811030985	0.071246799	0	0	1
ENSMUSG00000046598	Bdh1	-3.815899688	0.071006766	3.36E-208	1.01E-206	1
ENSMUSG00000033998	Kcnk1	-3.82868034	0.070380504	0	0	1
ENSMUSG00000051727	Kctd14	-3.831731751	0.070231801	0	0	1
ENSMUSG00000028435	Aqp3	-3.848389314	0.069425558	2.53E-56	2.10E-55	1
ENSMUSG00000026415	Fcamr	-3.852171929	0.069243769	1.70E-78	1.96E-77	1
ENSMUSG00000022150	Dab2	-3.858657671	0.068933177	0	0	1
ENSMUSG00000035686	Thrsp	-3.876384802	0.068091343	0	0	1
ENSMUSG00000043629	1700019D03Rik	-3.878784905	0.067978159	6.77E-262	2.57E-260	1
ENSMUSG00000043782	Ccdc64b	-3.88703454	0.067590554	2.64E-111	4.29E-110	1
ENSMUSG00000064356	mt-Atp8	-3.891225267	0.067394503	0	0	1
ENSMUSG00000020614	Fam20a	-3.891742649	0.067370338	0	0	1
ENSMUSG00000022270	Fam134b	-3.934890593	0.065385267	0	0	1
ENSMUSG00000027459	Fam110a	-3.942914955	0.0650226	0	0	1
ENSMUSG00000010601	Apol7a	-3.976692966	0.0635179	4.24E-105	6.47E-104	1
ENSMUSG00000040010	Slc7a5	-3.985014944	0.063152561	0	0	1
ENSMUSG00000011034	Slc5a1	-3.985678074	0.06312354	5.49E-192	1.53E-190	1
ENSMUSG00000073906	Olfr692	-3.988771193	0.062988349	1.98E-11	5.17E-11	1
ENSMUSG00000039552	Rsph4a	-3.995634039	0.062689427	7.41E-100	1.07E-98	1
ENSMUSG00000053461	Hhipl2	-4.020851018	0.061603195	7.75E-253	2.82E-251	1
ENSMUSG00000022309	Angpt1	-4.021460664	0.061577168	1.93E-196	5.49E-195	1
ENSMUSG00000022479	Vdr	-4.031295419	0.061158828	0	0	1
ENSMUSG00000022885	St6gal1	-4.041462695	0.060729332	0	0	1
ENSMUSG00000030428	Ttyh1	-4.051263737	0.060318161	0	0	1
ENSMUSG00000070511	Gm10295	-4.057400104	0.060062149	1.71E-105	2.62E-104	1
ENSMUSG00000024526	Cidea	-4.062286437	0.059859065	0	0	1
ENSMUSG00000011179	Odc1	-4.068113349	0.059617788	0	0	1
ENSMUSG00000009633	G0s2	-4.069346057	0.059566869	0	0	1
ENSMUSG00000019852	Arfgef3	-4.070986534	0.059499175	2.61E-199	7.58E-198	1
ENSMUSG00000029802	Abcg2	-4.081081055	0.059084313	0	0	1
ENSMUSG00000062542	Syt9	-4.114130914	0.057746171	7.32E-168	1.78E-166	1
ENSMUSG00000002308	Cd320	-4.146983573	0.05644605	2.52E-177	6.47E-176	1
ENSMUSG00000032091	Tmprss4	-4.162132853	0.055856429	5.06E-97	7.11E-96	1

ENSMUSG00000028003	Lrat	-4.176567378	0.055300358	2.51E-191	6.98E-190	1
ENSMUSG00000040013	Fkbp6	-4.203639374	0.054272329	1.14E-176	2.92E-175	1
ENSMUSG00000020917	Acly	-4.216631637	0.053785771	0	0	1
ENSMUSG00000046410	Kcnk6	-4.218664943	0.05371002	0	0	1
ENSMUSG00000027186	Elf5	-4.228455064	0.053346778	0	0	1
ENSMUSG00000090460	Gm17511	-4.256732199	0.05231135	2.97E-83	3.61E-82	1
ENSMUSG00000044645	Gm7334	-4.276792655	0.051589002	3.16E-188	8.70E-187	1
ENSMUSG00000029234	Tmem165	-4.286649703	0.051237727	0	0	1
ENSMUSG00000028655	Mfsd2a	-4.288991391	0.051154629	1.28E-105	1.96E-104	1
ENSMUSG00000035189	Ano4	-4.310865359	0.050384879	3.41E-191	9.46E-190	1
ENSMUSG00000017868	Sgk2	-4.336877898	0.049484555	4.19E-39	2.55E-38	1
ENSMUSG00000025165	Sectm1a	-4.342487363	0.049292523	1.65E-85	2.08E-84	1
ENSMUSG00000040035	Disp2	-4.363951945	0.048564572	2.68E-119	4.66E-118	1
ENSMUSG00000032349	Elovl5	-4.366423606	0.048481441	0	0	1
ENSMUSG00000022863	Btg3	-4.367470591	0.04844627	2.14E-210	6.59E-209	1
ENSMUSG00000020142	Slc1a4	-4.374713527	0.048203659	2.02E-218	6.44E-217	1
ENSMUSG00000033684	Qsox1	-4.437279114	0.046157884	0	0	1
ENSMUSG00000051452	Gm11437	-4.448404936	0.045803291	3.29E-17	1.10E-16	1
ENSMUSG00000027808	Serp1	-4.449610639	0.045765028	0	0	1
ENSMUSG00000025176	Hoga1	-4.456080205	0.04556026	5.82E-131	1.12E-129	1
ENSMUSG00000052271	Bhlha15	-4.460981032	0.045405755	0	0	1
ENSMUSG00000053897	Slc39a8	-4.480474241	0.044796374	0	0	1
ENSMUSG00000082766	1700064H15Rik	-4.491348566	0.04445999	8.98E-34	4.87E-33	1
ENSMUSG00000028772	Zcchc17	-4.493484656	0.04439421	0	0	1
ENSMUSG00000039236	Isg20	-4.513302361	0.043788555	6.77E-204	2.02E-202	1
ENSMUSG00000048776	Pthlh	-4.531418232	0.043242141	1.39E-63	1.28E-62	1
ENSMUSG00000073639	Rab18	-4.553380578	0.042588845	0	0	1
ENSMUSG00000020847	Rph3al	-4.602586144	0.041160772	0	0	1
ENSMUSG00000021125	Arg2	-4.65915462	0.039578078	4.05E-151	8.89E-150	1
ENSMUSG00000034112	Atp2c2	-4.684540448	0.038887749	0	0	1
ENSMUSG00000032204	Aqp9	-4.773195247	0.036570008	1.64E-45	1.14E-44	1
ENSMUSG00000028413	B4galt1	-4.820695132	0.035385568	0	0	1
ENSMUSG00000072618	Gm10384	-4.827681913	0.035214614	3.87E-43	2.56E-42	1
ENSMUSG00000025854	Fam20c	-4.843262525	0.034836355	0	0	1
ENSMUSG00000022797	Tfrc	-4.905077812	0.033375244	0	0	1
ENSMUSG00000010663	Fads1	-4.905085817	0.033375059	0	0	1
ENSMUSG00000050520	Cldn8	-4.968770222	0.031933838	0	0	1
ENSMUSG00000029755	Dlx5	-5.014435896	0.030938865	5.13E-28	2.42E-27	1
ENSMUSG00000090639	Gm20425	-5.016303804	0.030898834	0	0	1
ENSMUSG00000032554	Trf	-5.060498376	0.02996665	0	0	1
ENSMUSG00000037010	Apln	-5.095992273	0.029238391	1.04E-281	4.32E-280	1
ENSMUSG00000029304	Spp1	-5.115176537	0.028852166	0	0	1
ENSMUSG00000053216	Btn2a2	-5.116636398	0.028822986	1.61E-55	1.32E-54	1
ENSMUSG00000026955	Sapcd2	-5.121081343	0.028734319	1.18E-139	2.44E-138	1
ENSMUSG00000031936	Hephl1	-5.242399974	0.026416809	0	0	1
ENSMUSG00000028518	Prkaa2	-5.244523258	0.026377959	0	0	1
ENSMUSG00000031937	Vstm5	-5.285161393	0.025645306	2.62E-74	2.84E-73	1
ENSMUSG00000009378	Slc16a12	-5.330884813	0.024845273	9.23E-225	3.00E-223	1
ENSMUSG00000024558	Mapk4	-5.34684116	0.024571996	1.20E-215	3.78E-214	1
ENSMUSG00000003355	Fkbp11	-5.384822714	0.023933535	1.67E-131	3.24E-130	1
ENSMUSG00000032494	Tdgf1	-5.40391496	0.023618891	1.06E-29	5.24E-29	1
ENSMUSG00000006567	Atp7b	-5.458453396	0.022742688	3.65E-122	6.53E-121	1
ENSMUSG00000001827	Folr1	-5.497185454	0.022140238	0	0	1
ENSMUSG00000026417	Pigr	-5.60737555	0.020512177	0	0	1
ENSMUSG00000054169	Ceacam10	-5.641646564	0.020030655	3.95E-136	7.90E-135	1
ENSMUSG00000038094	Atp13a4	-5.678668857	0.01952317	0	0	1
ENSMUSG00000062380	Tubb3	-5.766531423	0.018369658	1.76E-99	2.52E-98	1

ENSMUSG00000000706	Btn1a1	-5.803422657	0.017905881	0	0	1
ENSMUSG00000056073	Grik2	-5.839748775	0.017460652	2.85E-82	3.40E-81	1
ENSMUSG00000040055	Gjb6	-5.859929285	0.017218111	5.39E-57	4.52E-56	1
ENSMUSG00000020617	1700012B07Rik	-5.988802498	0.015746746	3.41E-25	1.49E-24	1
ENSMUSG00000081906	Rpl9-ps1	-5.988933447	0.015745316	3.38E-17	1.13E-16	1
ENSMUSG00000022991	Lalba	-6.169315317	0.01389476	0	0	1
ENSMUSG00000042784	Muc1	-6.18939478	0.013702712	0	0	1
ENSMUSG00000050808	Muc15	-6.19578632	0.013642139	0	0	1
ENSMUSG00000028017	Egf	-6.197926253	0.013621919	0	0	1
ENSMUSG00000029695	Aass	-6.206846596	0.013537953	1.11E-78	1.28E-77	1
ENSMUSG00000026818	Cel	-6.268918798	0.012967833	0	0	1
ENSMUSG00000009588	St6galnac1	-6.310871194	0.012596169	2.93E-30	1.46E-29	1
ENSMUSG00000021187	Tc2n	-6.44108812	0.011509045	0	0	1
ENSMUSG00000022491	Glycam1	-6.495534293	0.011082796	0	0	1
ENSMUSG00000056366	Fabp3-ps1	-6.506315415	0.011000284	0	0	1
ENSMUSG00000021553	Slc28a3	-6.5428325	0.010725342	0	0	1
ENSMUSG00000009356	Lpo	-6.547620749	0.010689804	1.76E-145	3.76E-144	1
ENSMUSG00000028194	Ddah1	-6.554248063	0.010640811	1.14E-261	4.31E-260	1
ENSMUSG00000028773	Fabp3	-6.563355415	0.01057385	0	0	1
ENSMUSG00000067261	Foxd3	-6.689376314	0.009689404	1.17E-55	9.58E-55	1
ENSMUSG00000030732	Chrdl2	-6.868358957	0.0085589	0	0	1
ENSMUSG00000090688	Vmn2r12	-6.87647361	0.008510894	2.37E-44	1.61E-43	1
ENSMUSG00000061762	Tac1	-6.964714517	0.008005934	4.36E-68	4.31E-67	1
ENSMUSG00000047797	Gjb1	-7.035597111	0.007622093	4.46E-91	5.92E-90	1
ENSMUSG00000063129	Aldoart2	-7.339302906	0.006175181	7.94E-53	6.19E-52	1
ENSMUSG00000091450	Vmn2r11	-7.51248971	0.005476653	1.04E-45	7.26E-45	1
ENSMUSG00000029188	Slc34a2	-7.799065063	0.004490012	0	0	1
ENSMUSG00000024827	Gldc	-7.831490828	0.004390221	0	0	1
ENSMUSG00000001622	Csn3	-7.947737554	0.00405035	0	0	1
ENSMUSG00000068614	Actc1	-8.123686141	0.003585311	1.71E-100	2.50E-99	1
ENSMUSG00000021795	Sftpd	-8.176953715	0.003455347	6.76E-75	7.40E-74	1
ENSMUSG00000074715	Ccl28	-8.208597396	0.003380383	0	0	1
ENSMUSG00000063157	Csn2	-8.455893513	0.002847885	0	0	1
ENSMUSG00000026065	Slc9a4	-8.47128142	0.00281767	1.18E-123	2.16E-122	1
ENSMUSG00000015652	Steap1	-8.472711726	0.002814878	1.14E-30	5.73E-30	1
ENSMUSG00000024903	Lao1	-8.474151074	0.002812071	0	0	1
ENSMUSG00000091375	Vmn2r15	-8.661330841	0.002469901	6.63E-17	2.20E-16	1
ENSMUSG00000028836	Slc30a2	-8.770254202	0.00229029	3.72E-87	4.75E-86	1
ENSMUSG00000091059	Vmn2r14	-9.022324331	0.001923135	2.56E-18	8.99E-18	1
ENSMUSG00000067010	Vmn2r10	-9.092087892	0.001832352	7.22E-35	4.04E-34	1
ENSMUSG00000026644	Acbd7	-9.178727305	0.001725551	5.87E-19	2.10E-18	1
ENSMUSG00000091635	Vmn2r13	-9.217664255	0.001679603	4.57E-19	1.64E-18	1
ENSMUSG00000070702	Csn1s1	-9.547223971	0.001336593	0	0	1
ENSMUSG00000070366	Ppapdc1a	-9.632737835	0.001259671	3.62E-11	9.33E-11	1
ENSMUSG00000091624	Vmn2r9	-9.713402219	0.001191172	1.08E-40	6.81E-40	1
ENSMUSG00000090538	Gm4775	-9.75336219	0.001158632	0.000291025	0.000470736	1
ENSMUSG00000073752	Gm10570	-10.07537839	0.000926849	0.000168816	0.000278032	1
ENSMUSG00000090961	Vmn2r8	-10.11811135	0.000899798	3.26E-12	8.83E-12	1
ENSMUSG00000030302	Atp2b2	-10.24397537	0.000824624	0	0	1
ENSMUSG00000061937	Csn1s2a	-10.38068548	0.000750071	0	0	1
ENSMUSG00000094499	Vmn2r-ps104	-10.41656069	0.000731649	9.79E-05	0.000164907	1
ENSMUSG00000000381	Wap	-10.50564692	0.000687836	0	0	1
ENSMUSG00000026645	Olah	-10.55397735	0.000665175	0	0	1
ENSMUSG00000079025	Gsdmc	-10.74377875	0.000583177	1.19E-13	3.46E-13	1
ENSMUSG00000096368	Olfr1328	-10.75342725	0.00057929	5.55E-05	9.57E-05	1
ENSMUSG00000098348	Btg1-ps2	-10.9233553	0.000514923	4.10E-05	7.15E-05	1
ENSMUSG00000036463	4930544G11Rik	-11.07540536	0.000463416	3.06E-05	5.39E-05	1

ENSMUSG00000066031	Gm13023	-11.52608727	0.00033908	1.38E-05	2.52E-05	1
ENSMUSG00000002240	Prr27	-12.12266304	0.000224241	4.60E-06	8.70E-06	1
ENSMUSG00000061388	Csn1s2b	-13.72914349	7.36404E-05	0	0	1
ENSMUSG00000025496	Drd4	-13.79051266	7.05736E-05	1.71E-07	3.54E-07	1

Supplementary Table S2. **Differentially regulated signaling pathways in the mammary glands between wildtype and transgenic mice.**

Pathway analysis performed using differentially regulated genes identified in RNA-Seq (see the Methods section). Pathways are ranked by the p values.

Differentially Regulated Pathways

KEGG Pathway	Count	P-Value
mmu05150:Staphylococcus aureus infection	22	3.25E-09
mmu04672:Intestinal immune network for IgA production	18	2.00E-07
mmu04514:Cell adhesion molecules (CAMs)	38	1.10E-06
mmu04640:Hematopoietic cell lineage	25	1.38E-06
mmu05340:Primary immunodeficiency	15	1.89E-06
mmu01212:Fatty acid metabolism	18	4.86E-06
mmu03320:PPAR signaling pathway	23	7.53E-06
mmu05323:Rheumatoid arthritis	23	1.16E-05
mmu05166:HTLV-I infection	52	1.20E-05
mmu04660:T cell receptor signaling pathway	26	2.42E-05
mmu04060:Cytokine-cytokine receptor interaction	46	3.81E-05
mmu04062:Chemokine signaling pathway	39	4.55E-05
mmu04650:Natural killer cell mediated cytotoxicity	25	8.34E-05
mmu04923:Regulation of lipolysis in adipocytes	17	1.00E-04
mmu04380:Osteoclast differentiation	28	1.01E-04
mmu04662:B cell receptor signaling pathway	19	1.31E-04
mmu04976:Bile secretion	19	1.59E-04
mmu04670:Leukocyte transendothelial migration	26	3.27E-04
mmu04512:ECM-receptor interaction	21	3.46E-04
mmu04974:Protein digestion and absorption	21	3.46E-04
mmu04610:Complement and coagulation cascades	19	3.97E-04
mmu00980:Metabolism of xenobiotics by cytochrome P450	17	4.32E-04
mmu04064:NF-kappa B signaling pathway	22	5.01E-04
mmu05321:Inflammatory bowel disease (IBD)	16	5.34E-04
mmu04920:Adipocytokine signaling pathway	18	5.93E-04
mmu05146:Amoebiasis	24	0.001152
mmu04510:Focal adhesion	36	0.001311
mmu05140:Leishmaniasis	16	0.001327
mmu00061:Fatty acid biosynthesis	7	0.001476
mmu04978:Mineral absorption	12	0.00181
mmu04145:Phagosome	31	0.002076
mmu05144:Malaria	13	0.002202
mmu04015:Rap1 signaling pathway	36	0.002355
mmu04611:Platelet activation	25	0.00248
mmu04970:Salivary secretion	17	0.003505
mmu00620:Pyruvate metabolism	11	0.004141
mmu00982:Drug metabolism - cytochrome P450	15	0.005048
mmu05322:Systemic lupus erythematosus	26	0.005649
mmu04151:PI3K-Akt signaling pathway	51	0.006289

mmu04612:Antigen processing and presentation	17	0.006666
mmu00480:Glutathione metabolism	13	0.007226
mmu05310:Asthma	8	0.007464
mmu00053:Ascorbate and aldarate metabolism	8	0.014697
mmu01040:Biosynthesis of unsaturated fatty acids	8	0.014697
mmu05152:Tuberculosis	28	0.016083
mmu04964:Proximal tubule bicarbonate reclamation	7	0.018275
mmu05205:Proteoglycans in cancer	31	0.018965
mmu00071:Fatty acid degradation	11	0.021483
mmu01130:Biosynthesis of antibiotics	32	0.022382
mmu05202:Transcriptional misregulation in cancer	26	0.024661
mmu04152:AMPK signaling pathway	21	0.027243
mmu00983:Drug metabolism - other enzymes	11	0.02787
mmu05133:Pertussis	14	0.03101
mmu05332:Graft-versus-host disease	11	0.031524
mmu00100:Steroid biosynthesis	6	0.035349
mmu05204:Chemical carcinogenesis	16	0.039304
mmu00601:Glycosphingolipid biosynthesis - lacto and neolacto series	7	0.040023
mmu04940:Type I diabetes mellitus	12	0.042293
mmu04668:TNF signaling pathway	18	0.042935
mmu04918:Thyroid hormone synthesis	13	0.044043
mmu04925:Aldosterone synthesis and secretion	15	0.045952
mmu00860:Porphyrin and chlorophyll metabolism	9	0.047816
mmu05416:Viral myocarditis	14	0.049388
mmu05330:Allograft rejection	11	0.049501
